# Single-cell spatial transcriptomics unravels the cellular landscape of abdominal aortic aneurysm

**DOI:** 10.1172/jci.insight.190534

**Published:** 2025-08-22

**Authors:** Guizhen Zhao, Chun-Seok Cho, Hongyu Liu, Yongha Hwang, Yichen Si, Myungjin Kim, Yongjie Deng, Yang Zhao, Chao Xue, Yanhong Guo, Lin Chang, Dogukan Mizrak, Bo Yang, Hyun Min Kang, Jifeng Zhang, Jun Hee Lee, Y. Eugene Chen

**Affiliations:** 1 Department of Internal Medicine, University of Michigan Medical School, Ann Arbor, Michigan, USA.; 2 Department of Pharmacological and Pharmaceutical Sciences, University of Houston College of Pharmacy, Houston, Texas, USA.; 3 Department of Molecular & Integrative Physiology, University of Michigan Medical School, Ann Arbor, Michigan, USA.; 4 Department of Biostatistics, University of Michigan School of Public Health, Ann Arbor, Michigan, USA.; 5 Department of Cardiac Surgery, University of Michigan Medical School, Ann Arbor, Michigan, USA.

**Keywords:** Cardiology, Vascular biology, Cardiovascular disease

## Abstract

Abdominal aortic aneurysm (AAA) is a life-threatening vascular disease with no effective pharmacological interventions. While single-cell transcriptomics has advanced our understanding of AAA, it lacks spatial context. Here, we employed Seq-Scope, an ultra-high-resolution spatial transcriptomic technology, to decipher the spatial landscape of angiotensin II–induced AAA in *Apoe^–/–^* mice. Our analysis revealed the heterogeneity of macrophages, fibroblasts, and smooth muscle cells (SMCs), with specific responses in different layers of the AAA tissue. SMCs in the inner layers showed associations with Mgp-expressing fibroblasts and GPNMB-expressing macrophages, whereas the outer layers had different dominant cell types. Notably, GPNMB-expressing macrophages were concentrated near SMCs in regions of severe elastic lamina damage. Immunofluorescent staining confirmed their colocalization, and scRNA-seq reanalysis independently validated the presence of GPNMB-high macrophages in AAA tissues, highlighting their involvement in inflammation and tissue remodeling. Moreover, we discovered that macrophage-derived soluble GPNMB induces SMC phenotypic switching, reducing contractile markers while increasing cytokines and metalloproteinases. This effect was partly mediated by CD44 signaling. These findings suggest that GPNMB-high macrophages contribute to AAA development by driving SMC dysfunction. This study highlights the importance of high-resolution spatial transcriptomics in complementing single-cell transcriptomics, offering valuable insights into molecular and cellular responses in the AAA microenvironment.

## Abstract 2

Seq-Scope spatial transcriptomics reveals distinct cell populations in AAA, identifying Gpnmb-expressing macrophages that influence smooth muscle cell behavior and play a role in aneurysm development.

## Graphical abstract

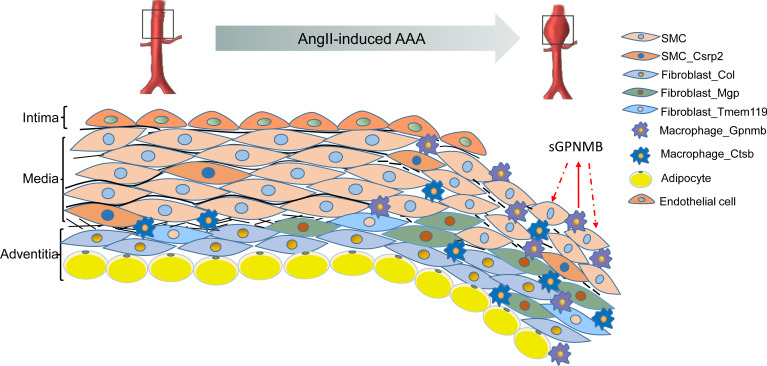

## Introduction

Abdominal aortic aneurysm (AAA) is a severe vascular disease with a high mortality rate upon rupture or dissection, making it the 13th leading killer in the United States (1). Numerous studies have identified vascular inflammation, smooth muscle cell (SMC) apoptosis and phenotypic switching, and extracellular matrix (ECM) degradation as the main pathological features of AAA (1–3). However, there is currently no proven pharmacological intervention for AAA. Recently, the application of single-cell RNA sequencing (scRNA-seq) has enabled profiling transcriptomic and cellular heterogeneity of human and murine AAA tissues at the single-cell level (4–7). A significant limitation of scRNA-seq, however, is its inability to preserve the spatial information of each cell in the original tissue. As a result, the connection between scRNA-seq findings and specific histopathology remains tenuous and speculative at best.

Glycoprotein nonmetastatic melanoma protein B (GPNMB), an endogenous type I transmembrane glycoprotein, has emerged as a key regulator in various pathological conditions, including vascular diseases (8–10). It is highly expressed by both resident and infiltrating tissue macrophages, with its soluble extracellular form produced through ectodomain shedding mediated by a disintegrin and metalloproteinase 10 (ADAM10), and has been implicated in inflammation, fibrosis, and tissue remodeling (8, 11–13). A recent study indicates that GPNMB is significantly elevated in angiotensin II–induced (AngII-induced) AAA mice, while metformin treatment inhibits this increase (10), suggesting its potential role in AAA progression. However, its precise contribution remains unclear. Investigating its cellular and spatial distribution as well function in AAA may offer new insights into disease mechanisms and potential therapeutic targets.

Spatial transcriptomics offers an attractive solution to this issue, as it can determine the location of endogenous transcripts using a barcoded spatial array. However, previous methodologies have faced resolution limitations, making single-cell-level analysis difficult. For example, the widely used 10× Visium has a 100 μm center-to-center distance, which is not suitable for profiling narrow, thin, and densely populated tissues such as aneurysmal aorta. In contrast, Seq-Scope, an integrated platform combining spatial barcoding and Illumina sequencing, provides a powerful approach for determining RNA expression and spatial information in individual cells, as well as the spatial patterning of cells within their native environment (14). Seq-Scope generates up to 1.5 million spatially defined barcode clusters in a 1 mm^2^ area with a center-to-center resolution of 0.5–0.8 μm, termed high-definition map coordinate identifier–array (HDMI-array). This high resolution enables visualization of transcriptomic heterogeneity at cellular and even subcellular levels in various tissues.

To define the tissue organization and spatial distributions of cellular responses in AAA, we generated the AngII-induced AAA model in *Apoe^–/–^* mice (15) and applied Seq-Scope to define how the transcriptomic landscape is spatially organized in the normal and aneurysmal aortic tissues, which would provide more insights to better understand the cell-specific molecular mechanism of AAA development.

## Results

### Seq-Scope analysis of normal and aneurysmal abdominal aortas.

**Figure 1:**
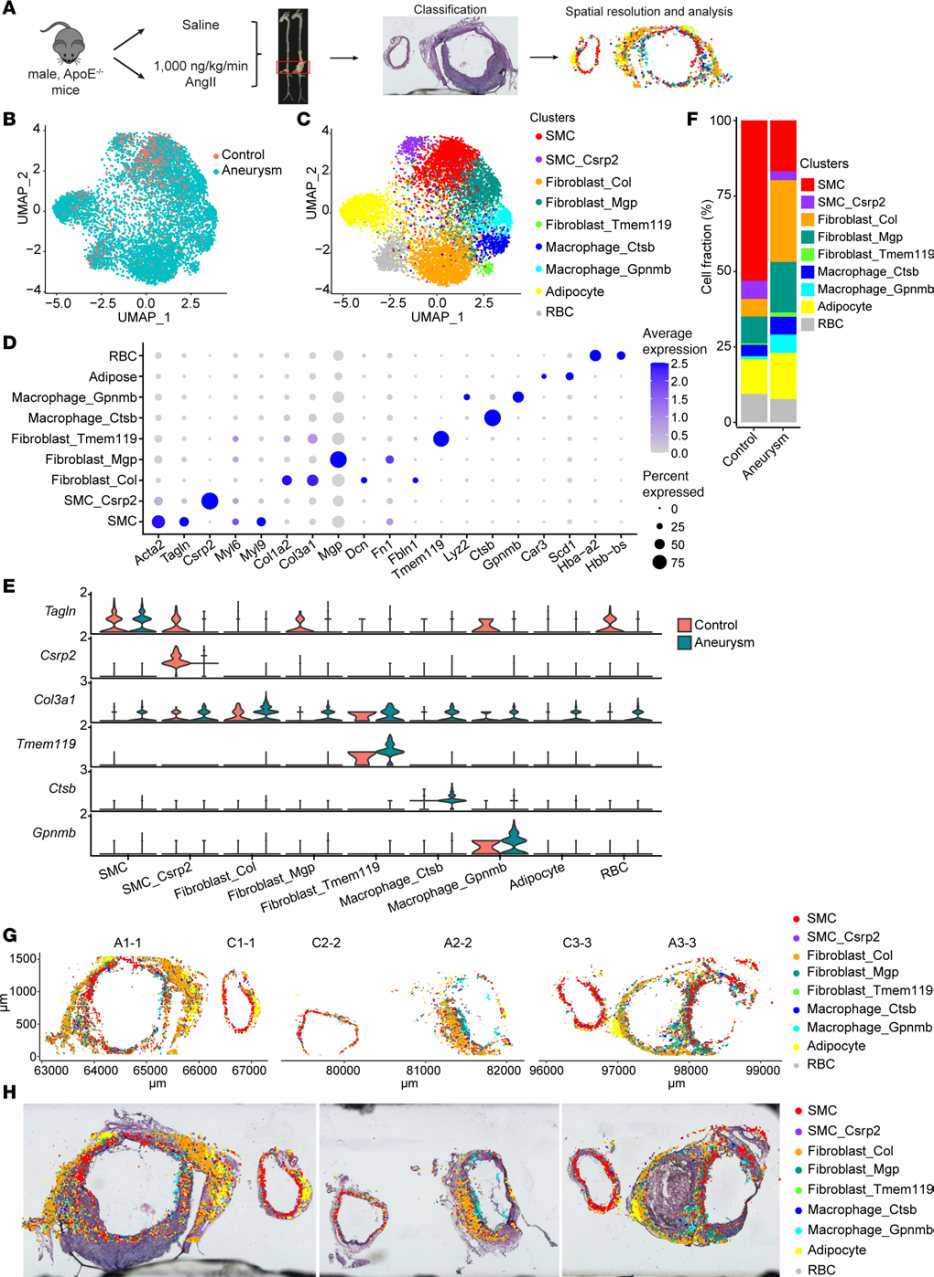
Single-cell spatial transcriptomic profiling of normal and aneurysmal abdominal aortas by Seq-Scope. Sixteen-week-old male *Apoe^–/–^* mice were infused with AngII (1,000 ng/kg/min) or saline via minipumps for 4 weeks. *n* = 5/group. (**A**) Schematic diagram depicting the mouse abdominal aortic aneurysm (AAA) and the spatial-transcriptomic analysis. (**B**) Uniform manifold approximation and projection (UMAP) of spatial transcriptomics spots from normal control and aneurysm group. The transcriptomics spots were from 10 sections of 3 normal control and 10 sections of 3 AAA group. (**C**) UMAP of cell-type clusters based on the gene compositions in each spatial transcriptomics spot. SMC, smooth muscle cells; Csrp2, SMC_Csrp2, SMCs highly expressing cysteine and glycine-rich protein 2; Fibroblast_Col, fibroblasts highly expressing collagen; Fibroblast_Mgp, fibroblasts highly expressing matrix Gla protein; Firboblast_Tmem119, fibroblast highly expressing transmembrane protein 119; Macrophage_Ctsb, macrophages highly expressing cathepsin B; Macrophage_Gpnmb, macrophages highly expressing glycoprotein Nmb; RBC, red blood cell. (**D**) Dot plot of marker genes for each cell population. (**E**) StackedVlnPlot showing the expression of marker genes across Control and Aneurysm groups. (**F**) Cell population percentages in each group. (**G** and **H**) Representative spatial plots of cell-type clusters (**G**) were overlaid with H&E imaging (**H**).

Lack of spatial information in the scRNA-seq studies severely limits our understanding of AAA pathology. To explore the pathogenic microenvironment of AAA, we applied Seq-Scope to characterize the spatial transcriptomic landscape of normal and aneurysmal aortas. Sixteen-week-old *Apoe^–/–^* male mice fed a normal diet were subcutaneously implanted minipumps to deliver AngII (1,000 ng/mL, 5 mice) or saline (5 mice) for 4 weeks (Figure 1A) (15). No significant difference was found in body weight, total cholesterol, and triglycerides between the 2 groups at 4 weeks after minipump implantation (Supplemental Figure 1, A and B; supplemental material available online with this article; https://doi.org/10.1172/jci.insight.190534DS1). The extraluminal diameters were significantly increased in AngII group, in which 3 mice developed AAA (60% incidence) (Supplemental Figure 1, C and D).

The fresh frozen suprarenal abdominal aortas from 3 mice with AAA and 3 saline-infused control mice were pair-embedded in optimal cutting temperature compound (OCT) and cut into 10-μm sections (Supplemental Figure 1, E, G, and I). A total of 20 aortic sections (10 normal aortic sections and 10 aneurysmal sections) were attached onto the HDMI-array slide for histological staining, library preparation, and sequencing (Supplemental Table 1). The spatial digital gene expression results were partitioned according to non-overlapping hexagons with 12 μm sides, and after applying 75 gene feature cutoffs, we obtained 104,762 hexagons. The hexagons with substantial gene expression information all corresponded to the areas under aortic sections (Supplemental Figure 1, F, H, and J). The hexagonal areas revealed 130.2 ± 55.6 unique transcripts and 115.6 ± 43.8 gene features. After data normalization as described in the Methods, we clustered the spatial data containing both groups (control and aneurysm, 10 sections/group) in an unsupervised manner using a graph-based approach, which identified 12 clusters (Supplemental Figure 2, A and B). To put the spots into context and assess their spatial organization, spots were projected onto the bright-field images of the same section stained with H&E (Supplemental Figure 1, E–G, and Supplemental Figure 2C). To further characterize the identified 12 clusters, we performed differential gene expression analysis among them. Cluster 0, which is located at the center of the uniform manifold approximation and projection (UMAP) did not show any substantial gene enrichment and did not show specific spatial localization (Supplemental Figure 2C), suggesting that this cluster represents a mixture of several cell types. Only a few genes, which were not suggestive of any known biological functions or cellular pathways, were detected for clusters 10 and 11. Identities of all clusters other than clusters 0, 10, and 11 were clear in biological pathways, so we focused our analyses on these annotatable clusters, including 2 SMC clusters: SMC (highly expressing contractile marker genes Acta2 [actin α2, smooth muscle] and Tagln [transgelin]) (7) and SMC_Csrp2 (enrichment of Csrp2 [cysteine and glycine rich protein 2]) (16); 3 fibroblast clusters: Fibroblast_Col (collagen-producing fibroblasts), Fibroblast_Mgp (Mgp-high [matrix Gla protein–high] fibroblasts) (17), and Fibroblast_Tmem119 (fibroblasts highly expressing Tmem19 [transmembrane protein 119]); 2 macrophage clusters: Macrophage_Ctsb (Ctsb-high [cathepsin B–high] macrophages) (18) and Macrophage_Gpnmb (Gpnmb-high macrophages) (19); 1 adipocyte cluster; and 1 red blood cell (RBC) cluster (Figure 1, B–F, and Supplemental Table 2). All of these clusters were found in all samples of AAA and normal controls (Figure 1B and Supplemental Figure 2D), and AAA and normal control samples overlapped with each other fairly well (Figure 1B), indicating that the batch-dependent effects of animal and experimental variations are minimal. These are consistent with the previous reports about vascular cell heterogeneity in AAA (5, 7, 20).

The identified cell populations showed specific cell fraction and spatial distribution in the normal and aneurysmal aortas (Figure 1, F–H, and Supplemental Figure 2D). Vascular SMCs (VSMCs) are the main cellular component in the normal aorta (5–7), accounting for more than 50%, and mainly located in the medial layer (Figure 1, F–H, and Supplemental Figure 2D). We also observed a decrease in VSMCs and an increase in macrophages, particularly the Macrophage_Gpnmb subpopulation, in AAA (Figure 1F and Supplemental Figure 2D), consistent with previous reports in both human and murine AAA (4, 5, 7).

Notably, compared with the normal control group, AAA exhibited an increased percentage of fibroblasts (Figure 1F and Supplemental Figure 2D), a unique pathological feature of AngII-induced AAA (20, 21). In the AAA tissues, all fibroblast clusters were localized in the adventitia but still showed different spatial distribution (Figure 1, G and H). Fibroblast_Col and Fibroblast_Tmem119 highly expressed Col3a1 (encoding α1 chain of type III collagen) and Col1a2 (encoding α2 chain of type I collagen), and were mainly positioned the adventitia far away from the medial layer, while Fibroblast_Mgp were present closely to the medial layer of small AAA and concentrated around the aortic area with dissection, identified from H&E histology images, spatial prediction, and factor inference of cartographic transcriptome at ultra-high resolution (FICTURE) (22) analysis (Figure 1, D, E, G, and H, Supplemental Figure 3A, Supplemental Figure 4, and Supplemental Table 2). Importantly, Fibroblast_Col and Fibroblast_Mgp highly expressed Fbln1 (encoding fibulin) and Fn1 (encoding fibronectin), respectively (Figure 1D and Supplemental Table 2), two ECM genes involved in matrix organization and tissue repair (23, 24). Moreover, these 2 fibroblast subpopulations were closely localized with Macrophage_Ctsb and Macrophage_Gpnmb, respectively, in the aneurysmal wall (Figure 1H, Supplemental Figure 3, B–E, and Supplemental Figure 4). These findings suggest that Fibroblast_Col and Fibroblast_Mgp may play important roles in AngII-induced AAA progression through ECM remodeling and immune system regulation.

### GPNMB-high macrophages are closely localized with medial VSMCs.

**Figure 2:**
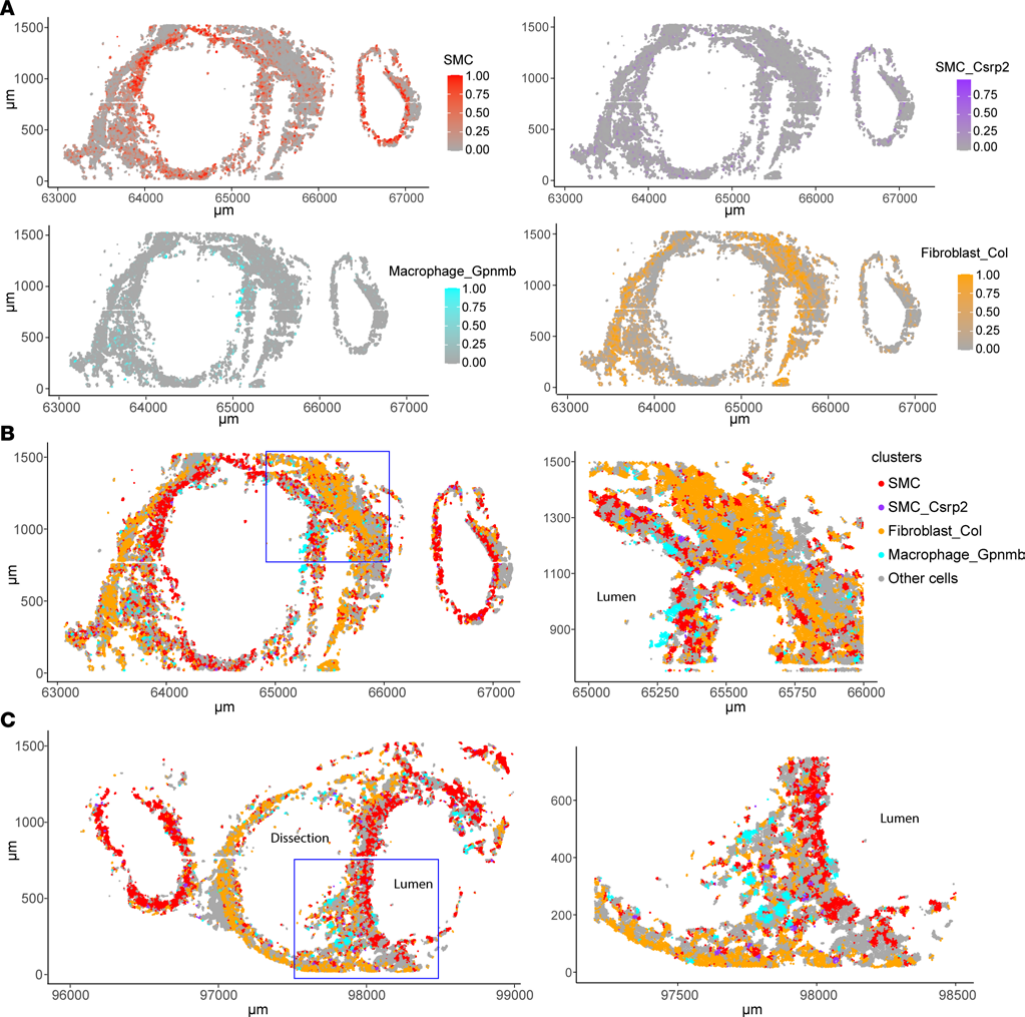
Spatial mapping of macrophages, GPNMB-high macrophages, and SMCs in normal aorta and AAA. (**A**) Spatial plot showing the spatial prediction score of SMC, SMC_Csrp2, Macrophage_Gpnmb, and Fibroblast_Col in the aortic tissues. (**B** and **C**) Spatial plot visualizing locations of SMC, SMC_Csrp2, Macrophage_Gpnmb, and Fibroblast_Col in the tissue sections. The boxed areas (blue boxes) are magnified on the right.

One of the key findings in this study is the identification of a macrophage subpopulation that highly expresses Gpnmb. Spatial prediction analysis further confirmed the spatial locations of SMC, SMC_Csrp2, Macrophage_Gpnmb, and Fibroblast_Col (Figure 2A). Of note, the majority of GPNMB-high macrophages were present in the medial layer in the AAA tissues and closely localized with SMCs, especially in the aortic tissue with dissection. Macrophage_Gpnmb were also increased in the adventitia, where external elastic layers were destructed (Figure 2, B and C). Additionally, the expression of Gpnmb in macrophages was markedly increased in AAA compared with that in normal group (Figure 1E).

**Figure 3:**
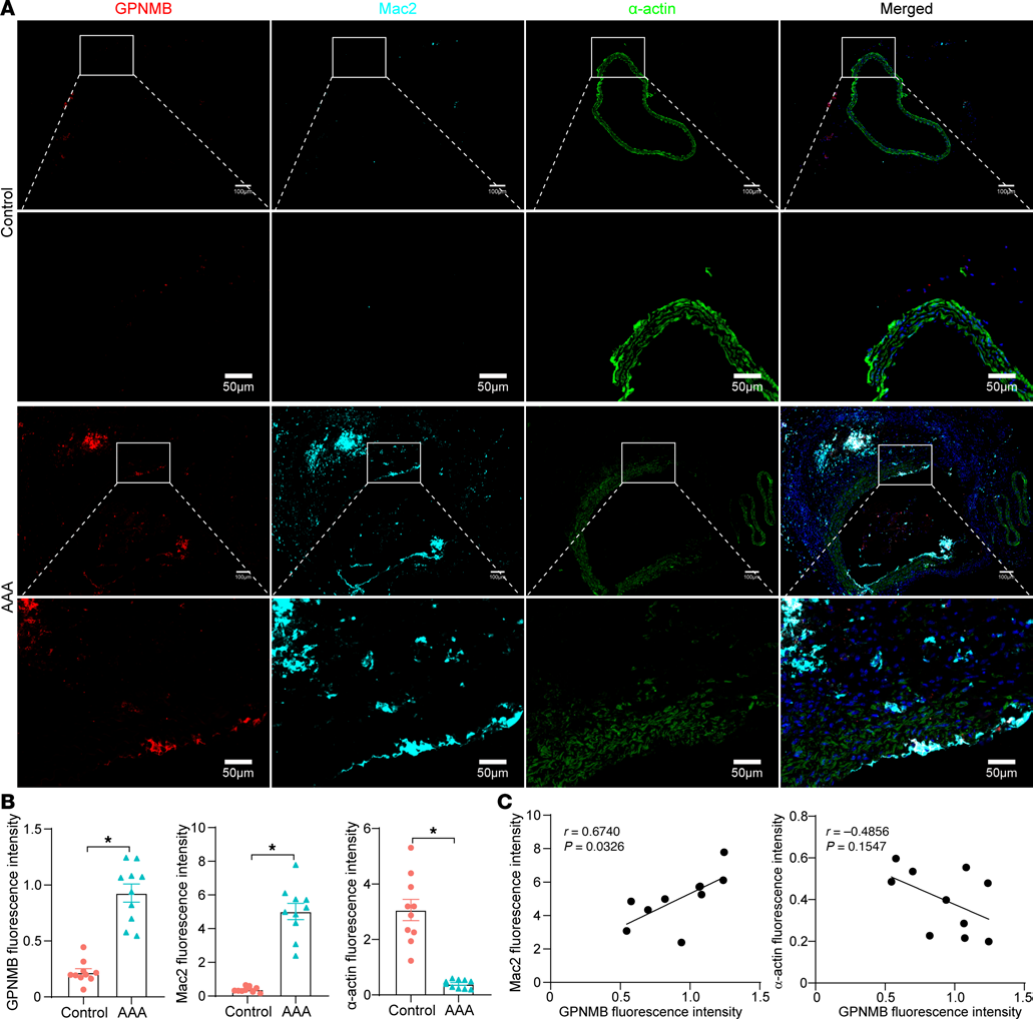
Immunofluorescent staining confirms the interaction of SMC and GPNMB-high macrophages in the aneurysmal aorta. (**A**) Immunofluorescent staining of GPNMB (red), Mac2 (cyan), and α-actin (green) to respectively show SMCs, macrophages, and GPNMB^+^ cells in the aortic wall of control and AAA. Nuclei stained with DPAI are blue. Scale bars: 100 μm (whole sections) and 50 μm (higher magnification areas). (**B**) Quantification of the GPNMB, Mac, and α-actin fluorescence intensity. (**C**) Correlation analysis between GPNMB and Mac2 or α-actin in AAA groups. Data are presented as mean ± SEM. *P < 0.05 by 2-tailed Student’s *t* test.

To independently confirm these observations through orthogonal technology, we performed immunofluorescent staining for GPNMB, a macrophage marker (Mac2), and an SMC marker (α-actin) in the supernal abdominal aortas of AAA and control group from an independent experiment (Figure 3A). GPNMB was mainly expressed in macrophages and significantly increased in the aneurysmal aorta (Figure 3A). Importantly, GPNMB^+^macrophages were frequently observed in close proximity to α-actin^+^ SMCs in the aortic wall (Figure 3, A and B). These results support observations from the Seq-Scope data (Figure 2B). However, a discrepancy between spatial transcriptomic and immunofluorescence data was observed regarding the location of GPNMB-high macrophages in the intima (Figure 2, B and C, and Figure 3, A and B). This may be attributed to potential loss or damage during tissue preparation in Seq-Scope analysis. Nevertheless, these findings are complementary and highlight the challenges in capturing the full spatial complexity of AAA lesions with different techniques. Furthermore, quantitative analysis revealed that GPNMB expression was significantly increased in AAA tissues and positively correlated with Mac2^+^ macrophages in the AAA group (*r* = 0.6740, *P* = 0.0326). Consistent with previous reports, SMC marker α-actin was significantly reduced in the AAA tissues and negatively correlated with GPNMB expression in the AAA group, despite no statistical significance (*r* = –0.4856, *P* = 0.1547). Together, these findings suggest that GPNMB-high macrophages may contribute to SMC dysfunction during AAA development.

### Macrophage-derived GPNMB induces SMC phenotypic switching.

**Figure 4:**
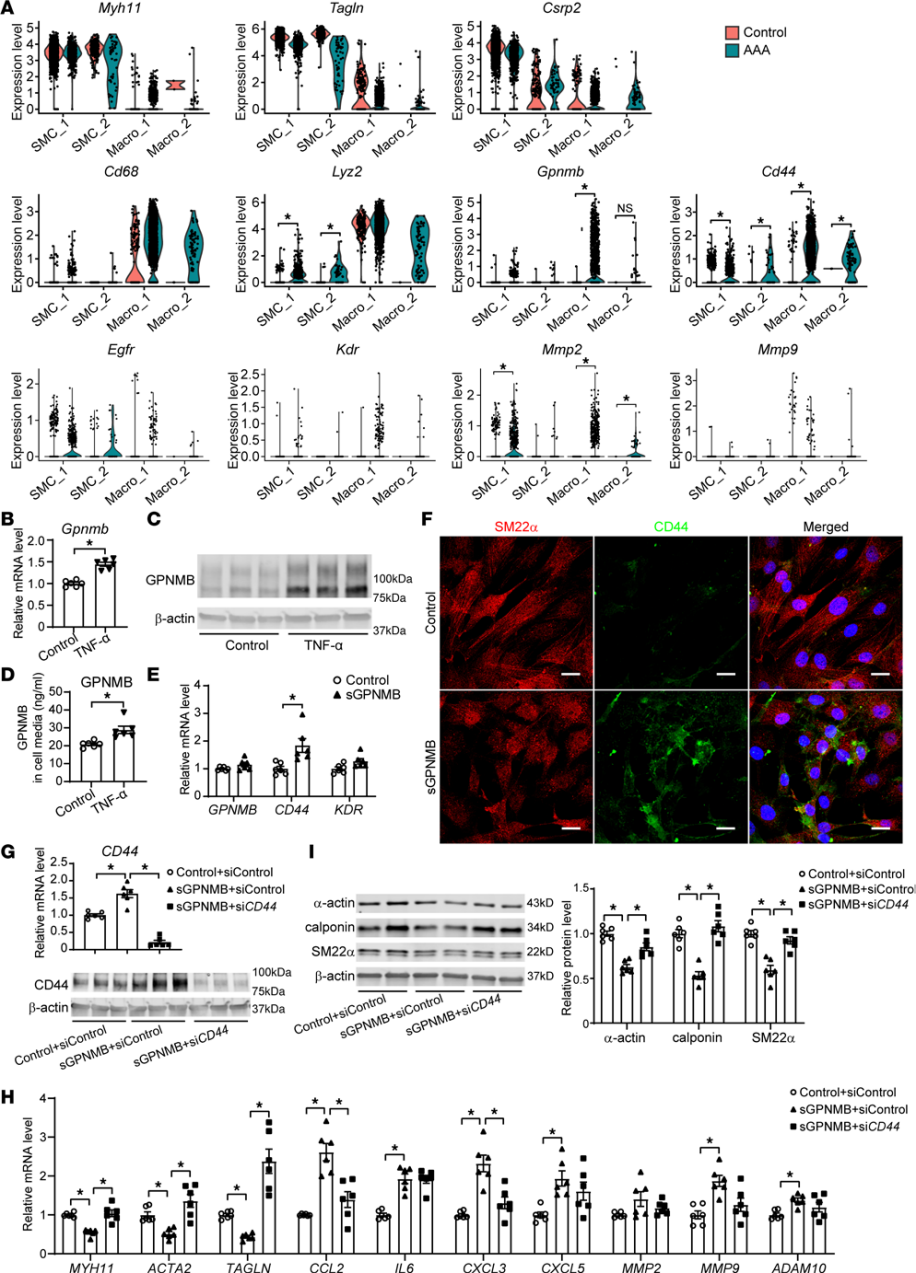
Macrophage-secreted GPNMB promotes SMC phenotypic switching. (**A**) Violin plot of *Myh11*, *Tagln*, *Csrp2*, *Cd68*, *Lyz2*, *Gpnmb*, *Cd44*, *Egfr*, *Kdr*, *Mmp2*, and *Mmp9* expression in SMC and macrophage subpopulations. The results were reanalyzed from scRNA-seq data (GSE193265 and GSE191226). The single cells were from healthy suprarenal abdominal aorta (Control) and abdominal aortic aneurysm (AAA) isolated from male *ApoE^–/–^* mice. (**B**–**D**) BMDMs isolated from C57BL/6J mice were stimulated with or without 10 ng/mL TNF-α for 24 hours. (**B** and **C**) Gpnmb mRNA and protein levels were determined. (**D**) The GPNMB protein abundance in the culture media was determined by ELISA (*n* = 6). (**E** and **F**) Human aortic smooth muscle cells (HASMCs), at 80% confluence, were serum starved for 24 hours and then treated with 40 ng/mL recombinant GPNMB (aa 22–486, sGPNMB) protein in fresh Opti-MEM for 48 hours. (**E**) qPCR was performed to determine the mRNA levels of *GPNMB*, GPNMB receptors *CD44* and *KDR*, and protease *ADAM10* (*n* = 6). (**F**) Immunofluorescent staining of SM22α and CD44. Nuclei stained with DPAI are blue. Scale bars: 20 μm. (**G**–**I**) HASMCs were transfected with nontargeting control siRNA (siControl) or si*CD44*. After 24 hours, the cells were serum starved for 24 hours and then treated with sGPNMB for 48 hours. qPCR was performed to determine the mRNA levels of *CD44* (**G**, upper panel, *n* = 6), SMC contractile markers *MYH11*, *ACTA2*, and *TAGLN* and inflammatory cytokines *CCL2* and *IL6* as well as proteases *MMP2*, *MMP9*, and *ADAM10* (**H**, *n* = 6). The protein abundance of CD44 (**G**, lower panel, *n* = 3) smooth muscle α-actin, calponin, and SM22α (**I**, *n* = 6) was determined by Western blot. Data are presented as mean ± SEM. Wilcoxon’s test was used for comparing means in **A**, 2-tailed Student’s *t* test for **B**, **D**, and **E**, and 1-way ANOVA followed by Tukey’s post hoc test for **G**–**I**. *P < 0.05.

To investigate the potential relationship between GPNMB-high macrophages and SMCs, we reanalyzed the published scRNA-seq dataset of AngII-induced AAA (6) (Supplemental Figure 5). Similarly, 2 SMC subpopulations (SMC_1, SMC_2) and 2 macrophage subpopulations (Macro_1, Macro_2) were clustered in the scRNA-seq dataset (Supplemental Figure 5A). Specifically, Gpnmb was highly expressed in the Macro_1 subpopulation, with higher expression of macrophage marker Cd68 (Supplemental Figure 5C). Gpnmb expression was significantly increased in AAA compared with that in the normal control group (Figure 4A). Consistently, reanalysis of human AAA scRNA-seq datasets (4, 25) revealed a similar macrophage subpopulation exhibiting elevated GPNMB expression in AAA lesions (Supplemental Figure 6). Notably, GO and KEGG enrichment analyses of the gene sets of the macrophage subpopulations revealed the functional similarities between Macrophage_Gpnmb and Macro_1 in both mouse and human scRNA-seq data, in particular pathways related to neutrophil degranulation, lysosome, leukocyte activation/regulation of cell activation, regulation of cytokine/TNF production, and inflammatory response (Supplemental Figure 7). These findings suggest a potential role of Gpnmb-high macrophages in modulating inflammation and tissue remodeling in AAA. These scRNA-seq analyses independently confirm the findings from Seq-Scope in that AAA-induced macrophage population is highly heterogeneous and that Gpnmb-high macrophages are substantial in AAA-induced macrophage populations.

Both spatial plot and immunofluorescence imaging showed that the colocalization between SMCs and Macrophage_Gpnmb was mainly observed in aortic sites with severe destruction of elastic layers (Figure 2B and Figure 3). GPNMB can be cleaved by metalloproteinases to produce the soluble GPNMB (sGPNMB), which can bind multiple receptors, including CD44 (26), epidermal growth factor receptor (EGFR) (27), kinase insert domain receptor (KDR), and integrin (28), to activate downstream signaling pathways. To clarify how macrophage-produced GPNMB affects VSMC function in AAA, we first analyzed the expression of Cd44, Egfr, Kdr, Mmp2, and Mmp9 in SMC and macrophage populations from an scRNA-seq dataset (6). Specifically, the SMC_1 subpopulation, which had a high level of Csrp2, showed a significant increase in the expression of Cd44 and Mmp2 in the AAA group (Figure 4A). Similarly, elevated expression of CD44 and MMP2 was identified in an SMC subpopulation within the AAA lesion in the human scRNA-seq data (Supplemental Figure 6C). Next, we examined the expression of GPNMB in mouse bone marrow–derived macrophages (BMDMs). As shown in Figure 4, B and C, GPNMB was upregulated at both mRNA and protein levels in response to TNF-α. Furthermore, sGPNMB was significantly increased in the culture media, as measured by ELISA (Figure 4D).

sGPNMB has been reported to facilitate ECM production in vascular fibroblasts by binding to integrin αVβ1 (28). To further explore whether sGPNMB affects VSMC phenotype or functional changes in AAA, sGPNMB (aa 22–486, 40 ng/mL) was used to treat human aortic SMCs (HASMCs). We found that sGPNMB significantly increased the expression of CD44, rather than KDR, in HASMCs (Figure 4E), consistent with the scRNA-seq data in Figure 4A. VSMCs are the predominant cell type within the aortic wall and its dysfunction, including apoptosis, phenotypic switching, and enhanced elastolysis, contributes to AAA development and progression (1–3, 29, 30). Importantly, immunofluorescent staining showed that sGPNMB increased CD44 and reduced the α-actin–containing stress fibers in HASMCs (Figure 4F), indicating a loss of VSMC contractile phenotype. Accordingly, the expression of VSMC contractile markers MYH11, ACTA2 (encoding α-actin), and TAGLN (encoding SM22α) was significantly decreased, whereas the proinflammatory cytokines CCL2, IL6, CXCL3, and CXCL5 were markedly increased in sGPNMB-treated HASMCs (Figure 4, H and I). Consistent with this, in SMC-specific differential expression assays using the Seq-Scope dataset, we were able to determine that many contractile markers, such as Acta2, Myl6 and Myl9, were strongly and significantly downregulated in the SMC clusters of AAA (Supplemental Table 3). These data suggest that sGPNMB suppresses VSMC contractile functionality and rather upregulates its proinflammatory characteristics. Besides, sGPNMB significantly enhanced the expression of metalloproteinases in HASMC, including matrix metalloproteinase 9 (MMP9) and a disintegrin and metalloproteinase 10 (ADAM10) (Figure 4H), which facilitate ECM degradation (30, 31).

CD44, EGFR, and integrins are common receptors that bind to GPNMB, and then trigger cellular responses, such as expression and secretion of MMPs, cytokines, and ECM production (28, 32–34). To further explore the role of CD44 in sGPNMB-induced SMC phenotypic switching, knockdown of CD44 was applied in HASMCs (Figure 4, G–I, and Supplemental Figure 8). We found that knockdown of CD44 can restore sGPNMB-induced reduction of VSMC contractile makers at both mRNA and protein levels (Figure 4, H and I). In addition, knockdown of CD44 also reduced sGPNMB-induced CCL2 and CXCL3 expression in HASMCs (Figure 4H). However, the increased expression of cytokines IL6, CXCL5, MMP9, and ADAM10 in sGPNMB-treated HASMCs could not be blocked by CD44 knockdown. Taken together, these findings suggest that sGPNMB secreted from macrophages causes VSMC phenotype switching and elastolysis, all of which contribute to the dilation and rupture of the aortic wall.

## Discussion

The molecular and cellular heterogeneity of vascular and immune cells in AAA have been widely profiled in recent scRNA-seq studies (4–7, 21). However, the spatial organization of these cells and cell-cell interaction within the aortic wall is lost due to tissue dissociation, which may also deplete vulnerable cell types and produce stress-associated transcriptional changes (35). In contrast, Seq-Scope (14), an ultra-high-resolution spatial barcoding technology, can monitor all cell types present in a tissue section with single-cell and subcellular spatial resolution, thus complementing single-cell transcriptomics approaches. Here, we applied Seq-Scope to investigate an AngII-induced AAA model and profiled the spatial transcriptomic landscape of normal and aneurysmal aortic tissues.

AngII infusion, perivascular application of calcium, and elastase are the 3 most commonly used mouse AAA models (15, 36, 37). All these models enable recapitulation of the major pathological hallmarks of human AAA, including decreased SMCs and increased immune cells, which were also identified by scRNA-seq analyses (5, 7, 20). The AngII-induced AAA model is more comparable to human AAA in terms of VSMC apoptosis, ECM degradation, immune cell infiltration, collagen deposition, angiogenesis, and high-risk factors, such as sex, age, smoking, and hyperlipidemia (38). Several scRNA-seq studies have demonstrated the cellular heterogeneity of the AngII-induced AAA model (6, 20, 21). However, these studies identified varying proportions of VSMCs and fibroblasts, particularly in the healthy aorta, which may result from insufficient tissue dissociation or the loss of fragile cell types during the process (35). In contrast, spatial transcriptomics techniques preserve the spatial information of the cells and gene expression in the original tissues, thus complementing single-cell transcriptomics approaches.

Leveraging the spatial transcriptomic data, we obtained findings consistent with previous reports, yet revealed spatial insight that was undetectable from the scRNA-seq datasets. For instance, our work revealed the cellular heterogeneity of SMCs, fibroblasts, and macrophages, which is in line with former scRNA-seq datasets (5–7, 20, 21); however, we believe our dataset is unique in showing that the VSMCs are the major population in normal aortic tissue, an observation that is more consistent with histological findings. Therefore, with the Seq-Scope dataset, we are in a better position to more precisely examine the cellular components that are altered during AAA induction. AAA induction indeed induced a huge change in cellular components, with gross changes in histological layers. AAA induction leads to a significant reduction in VSMC proportion in the medial layer and an increase in macrophage accumulation in the aneurysmal aorta wall. These findings further validate the pathological features of AAA reported in histological and scRNA-seq studies (1, 2, 4–7, 20, 21, 39). Moreover, both previous studies and our spatial transcriptomic results demonstrated that fibroblasts were significantly increased and made up the largest population in the aortic tissues in AngII-induced AAA (20, 21), despite different proportions of fibroblasts in the normal aortas. The study by Weng et al. revealed a fibroblast subcluster that highly expressed Fn1 and Vim and was mainly localized around the thrombus (21). Meanwhile, this fibroblast cluster was demonstrated to be closely connected with VSMCs through histological staining and cell-cell communication analysis (21). Consistently, our data identified a fibroblast subpopulation, Fibroblast_Mgp, highly expressing Mgp and Fn1, that was mainly distributed in the dissected site of the aorta and close to medial VSMCs. Moreover, we observed a significant increase in Fibroblast-Col, which highly express collagen-related genes and Fbln1 and are mainly distributed in the adventitia, with more accumulation of Ctsb-expressing macrophage in the AAA. The consistent results from our and previous studies (20, 21) indicate the critical roles of fibroblasts in the pathogenesis of AngII-induced AAA. It is noteworthy that our study further revealed the spatial colocalization patterns between fibroblast and macrophage subpopulations in AAA tissues, which were not revealed in scRNA-seq studies. Moreover, it should be noted that some of the GPNMB-high macrophages were also closely localized with Fibroblast_Mgp in the medial layer of AAA tissue. Thus, these results indicated that GPNMB-high macrophages might also contribute to the transcriptomic and functional changes in fibroblasts, which are among the most conspicuous characteristics of the AngII-induced AAA model.

Macrophage infiltration into the aneurysmal aortic wall has important roles in AAA pathophysiology through its effects on inflammation, ECM degradation, and tissue remodeling (40). Prior scRNA-seq studies also demonstrated a significant increase in immune cells, particularly macrophages, in both human and mouse AAA tissues (4, 5, 7, 21, 25). Our spatial transcriptomic study further confirmed the increased macrophage accumulation across all 3 layers of the aneurysmal aortic wall. More importantly, we identified a macrophage subpopulation with high GPNMB expression, which was significantly increased in AAA tissues, consistent with published human and mouse scRNA-seq datasets (4, 6, 25). Regarding spatial distribution, both spatial mapping and immunofluorescence imaging revealed that GPNMB-high macrophages are predominantly localized in regions with severe elastic layer destruction and close proximity to VSMCs. This suggests a potential functional interaction between macrophages and VSMC in AAA pathogenesis. While previous scRNA-seq studies inferred cell-cell communication networks in experimental AAA models and human AAA (21, 41), based on known ligand-receptor interactions, they lacked direct spatial validation. By leveraging spatial transcriptomics, our study provides insights into the anatomical organization of healthy and aneurysmal aortas and reveals the direct spatial proximity between VSMCs and GPNMB-high macrophages in the aortic wall. These findings strongly support the potential for direct cell-cell interactions, although further functional studies are required to confirm their causal role in AAA progression.

Emerging evidence suggests that GPNMB is upregulated in proinflammatory and pathological conditions (11, 27, 42). Our study further demonstrates that TNF-α, a crucial contributor to AAA (43), significantly increased the expression and release of GPNMB in macrophages. GPNMB exists in both membrane-bound and soluble forms, with the latter being cleaved by metalloproteinase ADAM10 and capable of binding to receptors such as CD44, EGER, and integrins to regulate ECM remodeling, cytokine production, and MMP activation (28, 32–34). Our findings reveal that sGPNMB promotes SMC phenotypic switching, characterized by decreased contractile markers and increased proinflammatory cytokines and metallopeptidases, all of which contribute to AAA development. A particularly intriguing finding of our study is the increased expression of CD44 in aneurysmal VSMCs and in sGPNMB-treated HASMCs. CD44 has been reported to negatively regulate α-smooth muscle actin expression in fibroblasts via the G-actin/MRTF/SRF-dependent pathway (44), and MRTF and SRF are well-known regulators of VSMC contractile gene expression (45, 46). Consistently, we found that knockdown of CD44 significantly attenuates sGPNMB-induced suppression of contractile markers and partially restores cytokine expression in VSMCs. These results, along with prior studies, indicate that the GPNMB/CD44 signaling axis may play a novel role in the regulation of VSMC dysfunction in AAA pathogenesis. Beyond its role in promoting VSMC phenotypic modulation, sGPNMB also enhances the expression of MMP9 and ADAM10 in HASMCs, facilitating ECM degradation and elastolysis. Notably, while CD44 knockdown restored VSMC contractile markers and reduced some cytokines (CCL2, CXCL3), it did not block the upregulation of IL6, CXCL5, or metalloproteinases, suggesting other receptors or signaling axis may be involved.

Despite these significant insights, an important limitation of our study is that the aortic tissues analyzed were from 4-week AngII-infused ApoE-null mice, a stage at which severe AAA with marked loss of VSMCs is typically observed. As a result, our study does not capture the dynamic changes in GPNMB expression or its impact on VSMC phenotype throughout AAA progression. Future studies incorporating time-course analyses or alternative models representing earlier disease stages would be valuable for elucidating the temporal regulation of GPNMB and its role in AAA initiation and progression.

In conclusion, our study provides the first unbiased high-resolution analysis to our knowledge of the spatial transcriptome in healthy and aneurysmal aortas in mice, which reveals the tissue architecture associated with AAA with single-cell resolution. Through this analysis, we identified a macrophage subpopulation that highly expresses GPNMB and predominantly distributes in the adventitia and medial layer with severe elastic destruction, which has not been characterized by previous studies. In addition to capturing and supporting previous observations of cellular and functional heterogeneity in the AAA tissues, our study provides insights into how GPNMB-high macrophages exert their role in AAA both intracellularly and in a paracrine fashion. The conserved macrophage subpopulation expressing GPNMB across both mouse and human AAA tissues suggests the translational relevance of these findings. The GPNMB/CD44 axis emerges as a potential therapeutic target, but further studies are needed to clarify its role and explore targeted interventions. With expected future advances in the spatial transcriptomics field, the improvements in spatial-omics resolution and molecule annotation capability will promote detailed investigations of rare cell types in disease pathogenesis.

## Methods

### Sex as a biological variable.

Male mice were used in this study due to the higher incidence rate of AngII-induced AAA observed in males compared with females, as documented in previous studies (47, 48). Research suggests that sex hormones, particularly the protective effect of estrogen in females, may contribute to this male predominance (47, 48). While this study focused on males, the mechanism identified may be relevant to both sexes, and future studies are needed to explore potential sex-specific differences.

### Murine AAA model.

The AngII-induced AAA model was generated as previously described (3, 15). Briefly, 12-week-old male *Apoe^–/–^* mice (The Jackson Laboratory, stock 002052) were subcutaneously implanted with minipumps (Alzet, model 2004) to infuse AngII (1,000 ng/kg/min; Bachem, H-1706) or saline (control group) for 4 weeks. All mice were fed a standard diet. After 28 days of AngII infusion, mice were euthanized by an overdose of CO_2_ and perfused with saline through the left ventricle to remove blood from the aorta after blood collection. The maximal outer diameter of the suprarenal abdominal aorta was measured with a digital caliper (Thermo Fisher Scientific, 14-648-17) as an average of 3 measurements in a double-blinded manner. Suprarenal abdominal aortas with a maximal diameter greater than 50% larger than its adjacent portion were considered as AAA (29, 49). Serum total cholesterol and triglyceride levels were measured by enzymatic kits (Wako Diagnostics). The fresh suprarenal abdominal aortic tissues were embedded in OCT for histological and Seq-Scope analyses.

### Seq-Scope.

The experimental procedures were performed as we previously described (14), but it was adapted to the Illumina HiSeq 2500 flow cell system so that Seq-Scope has a wider and continuous imaging area (1.5 mm × 60 mm in the HiSeq 2500, compared with 1 mm-diameter circles in MISEQ) (50). A single imaging area was used for all 20 samples to minimize the experimental batch effects. In brief, HDMI32-Dral (seed HDMI-oligo, containing the PCR/read adaptor sequences, the restriction enzyme–cleavable RNA-capture domain [oligo-dT], the HDMI, which is a spatial barcode composed of a 32 random nucleotide sequence) was amplified on a flow cell surface to generate HDMI-oligo clusters. The HDMI sequence and coordinates of each cluster were determined using the HiSeq 2500 platform. Thumbnail images of clusters were visualized using Illumina Sequencing Analysis Viewer to inspect the cluster morphology and density. Next, the HiSeq 2500 flow cell was processed to expose the oligo-dT and RNA-capture domain and generate an HDMI-encoded RNA-capturing array (HDMI-array). Subsequently, the HiSeq 2500 flow cell was disassembled to expose the HDMI-array to the outside and used for tissue attachment. The OCT-mounted fresh frozen aortic tissue was sectioned 10 μm thickness in a cryostat (Leica, CM3050S; –20°C) and the aortic sections were maneuvered onto the HDMI-array slide. After fixing in 4% formaldehyde for 10 minutes and incubating in isopropanol for 2 minutes, the tissue was stained with hematoxylin (Agilent, S3309) for 5 minutes, treated with bluing buffer (Agilent, CS702) for 2 minutes, and incubated with buffered eosin (eosin [Sigma-Aldrich, HT110216] and 0.45 M Tris-acetate buffer, pH 6.0 at a 1:9 ratio). After mounting in 85% glycerol, the tissues were imaged under a digital imager (Keyence, BZ-X800).

Total RNAs released from the fixed tissues were processed as previously described (51). Briefly, the fixed tissues attached on the HDMI-array slide were treated with 0.2 U/μL collagenase I (Thermo Fisher Scientific, 17018-029) at 37°C for 20 minutes, and then incubated with 1 mg/mL pepsin (Sigma-Aldrich, P7000) in 0.1 M HCl at 37°C for 10 minutes. The RNAs released from the tissue sections were captured by the HDMI-array and used as a template to generate cDNA footprints on the HDMI-barcoded RNA capturing molecule by reverse transcription. After overnight reverse transcription reaction at 37°C, the tissues were incubated in exonuclease I cocktail (1 U Exo I enzyme [New England Biolabs, M2903] in 1× Exo I buffer) and subsequently digested in 1× tissue digestion buffer (100 mM Tris pH 8.0, 100 mM NaCl, 2% SDS, 5 mM EDTA, 16 U/mL proteinase K [New England Biolabs, P8107S]). After tissue digestion, the secondary strand, a chimeric molecule of HDMI and cDNA sequences, was synthesized on the cDNA footprint using an adaptor-tagged random primer (Truseq Read 2-conjugated Random Primer version 2 with TCAGACGTGTGCTCTTCCGATCTNNNNNNNNB sequence). After removing all DNAs that did not bind to the HDMI-array, the secondary strand product was eluted using NaOH, and after neutralization, purified using AMPure XP purification kit (Beckman Coulter, A63881). The secondary strand product was used as a template to construct libraries using Kapa HiFi Hotstart Readymix (KAPA Biosystems, KK2602) through 2 rounds of library PCR. After purification using Zymoclean Gel DNA Recovery Kit (Zymo Research, D4001) and AMPure XP, the pooled libraries were subjected to paired-end (100–150 bp) sequencing in the Illumina platform at Psomagen lnc.

### Quantification and visualization of spatial transcriptomic data.

Seq-Scope data were initially processed using the STtools package (52), utilizing STAR/STARsolo 2.7.5c (53), with a modification to use a hexagonal array instead of a square array. The processed digital gene expression matrix, pooled in non-overlapping hexagons with 12-μm sides, was analyzed in the Seurat v4 package (54). First, we removed all genes starting with mt and Gm to remove mitochondrial genes and hypothetical gene models. Then feature number <75 was removed, and data were normalized using regularized negative binomial repression implemented in Seurat’s SCTransform function. Clustering analysis was performed using the shared nearest neighbor modularity optimization implemented in Seurat’s FindClusters function. The clusters were visualized in the UMAP plot or the histological space using ggplot functions. The markers for each cluster were identified through the FindAllMarkers function and used to annotate cell types. The clusters identified from the initial non-overlapping hexagonal array were projected onto the multiscale sliding windows (MSSW) array, which has 4-times higher resolution compared with the original non-overlapping array (52), using FindTransferAnchors, TransferData, and GetTransferPredictions functions, so that we could achieve higher resolution for cell type predictions.

### BMDMs.

BMDMs were obtained as previously described (55). In brief, 6- to 8-week-old C57BL/6J mice were euthanized and femurs and tibias were collected. Bone marrow cells in the medullary cavity were washed out with PBS and then cultured in Iscove’s modified Dulbecco’s media (IMDM, Gibco) supplemented with 10% heat-inactivated FBS (Gibco), 1% sodium pyruvate (Gibco), 1% non-essential amino acids (Gibco), 1% penicillin/streptomycin solution (Gibco), and 30% L929 supernatant as a macrophage-stimulating factor. After 7 days, the adherent macrophages were scraped off and seeded in new cell culture plates for the following experiments. The differentiated BMDMs were cultured in DMEM supplemented with 10% FBS and 1% penicillin/streptomycin solution.

### Cell culture.

HASMCs (Lonza, CC-2571) were cultured in SMC growth medium 2 (Lonza, CC-3182) containing 5% FBS (Lonza) and 1% penicillin/streptomycin solution (Gibco, 15140122) at 37°C, 5% CO_2_ in a humidified cell culture incubator. HASMCs were used from passages 4 to 8 in all experiments.

### siRNA transfection.

HASMCs were transfected with 30 nM siCD44 (ThermoFisher Scientific, s225167) or Silencer Select Negative Control siRNA (siControl, ThermoFisher Scientific, 4390843) using Lipofectamine RNAiMAX Reagent (Invitrogen, 13778150) according to the manufacturer’s instructions.

### Total RNA isolation and quantitative real-time PCR analysis.

Total RNA from BMDMs, mouse SMCs, or HASMCs was extracted using an RNeasy Mini Kit (QIAGEN, 74106) according to the manufacturer’s instructions. SuperScript III First-Strand Synthesis System (Thermo Fisher Scientific, 18080051) and random primers were used to reverse transcribe RNA into cDNA. Gene expression was quantified by Real-Time PCR Detection System (Bio-Rad) using iQ SYBR Green Supermix (Bio-Rad, 1708882). The gene expression level was normalized to the internal control β-actin. The primer sequences used are listed in Supplemental Table 4.

### Quantification of GPNMB release from BMDMs by ELISA.

BMDMs were seeded at 5 × 10^5^ cells/well in 6-well plates and treated with 10 ng/mL TNF-α for 24 hours. GPNMB was released into the culture media and measured by using the mouse Osteoactivin/GPNMB DuoSet ELISA kit (R&D Systems, DY2330) in accordance with the manufacturer’s instructions.

### Protein extraction and Western blot.

Cells were lysed in RIPA lysis buffer (Thermo Fisher Scientific, 89901) supplemented with cOmplete EDTA-free protease inhibitor cocktail (Roche, 11873580001) and PhosSTOP phosphatase inhibitor (Roche, 4906845001). Cells were lysed at 4°C for 30 minutes and centrifuged at 12,000*g* for 15 minutes to remove insoluble debris. Protein extracts were resolved in 10% SDS-PAGE gels and transferred to nitrocellulose membranes (Bio-Rad, 1620115). Membranes were blocked in TBST containing 5% fat-free milk for 1 hour at room temperature and incubated with primary antibodies anti-SM22α (Abcam, ab10135) and anti–α-actin (Abcam, ab119952) at 4°C overnight. After washing 3 times with 1× TBST, membranes were incubated with secondary antibodies (1:10,000 dilution, Li-Cor Bioscience) for 1 hour at room temperature. After 3 washes with 1× TBST, fluorescent signals were scanned using the Odyssey Imaging System (Li-Cor Bioscience) and quantified with the LI-COR Image Studio Software.

### Immunofluorescent staining.

The aortic sections embedded in OCT were rehydrated and blocked with 5% donkey serum in PBS for 1 hour at room temperature. Next, the aortic sections were stained with primary antibodies anti-GPNMB (Thermo Fisher Scientific, AF2330), anti-Mac2 (Thermo Fisher Scientific, 14-5301-85), and anti–α-actin (Abcam, ab5694) at 4°C overnight.

HASMCs cultured in Falcon chambered cell culture slides (Thermo Fisher Scientific, 08774208) were fixed in 4% formaldehyde in PBS for 15 minutes at room temperature. After washing with PBS 3 times, slides were incubated in a permeabilizing solution (PBS with 0.1% Triton X-100 and 0.5% BSA) for 10 minutes and blocked with 5% donkey serum for 1 hour at room temperature. Then, slides were stained with primary antibodies anti-CD44 (Thermo Fisher Scientific, 5012580) and anti-SM22α (Abcam, ab10135) at 4°C overnight.

After washing with PBS, the aortic sections and HASMCs were incubated with Alexa Fluor–conjugated secondary antibodies (Jackson ImmunoResearch Laboratories) for 1 hour at room temperature. After washing with PBS, slides were mounted with ProLong Gold Antifade Mountant with DAPI (Invitrogen, P36935) before image collection with a Nikon A1si confocal microscope.

### Statistics.

GraphPad Prism 8.0 and RStudio software were used for statistical analyses. Data are presented as mean ± standard error of the mean (SEM). After normality and equal variance evaluation, Student’s *t* test was used for normally distributed data, while non-parametric tests were used for nonnormally distributed data to compare the difference between 2 groups. A *P* value of less than 0.05 was considered statistically significant.

### Study approval.

All animal procedures were performed according to protocols approved by the Institutional Animal Care & Use Committee (IACUC) at the University of Michigan and complied with the NIH *Guide for the Care and Use of Laboratory Animals* (National Academies Press, 2011).

### Data availability.

The Seq-Scope aorta dataset generated from this study is available at the Gene Expression Omnibus database (GEO) under accession number GSE229500. All quantitative data points underlying the graphs and statistical analyses are provided in the Supporting Data Values file. All other data supporting the findings of this study are included in the article and its supplemental material or are available from the corresponding authors upon reasonable request.

## Author contributions

GZ and CSC performed experiments. GZ, HMK, and JHL performed data analysis. YH, YS, and MK assisted with data analysis and interpretation. GZ and JZ wrote the manuscript. HL, YD, YZ, CX, YG, LC, DM, BY, and JZ provided technical support and contributed to the discussion of the project and manuscript. YEC, JZ, and JHL designed the research.

## Supplementary Material

Supplemental data

Unedited blot and gel images

Supporting data values

## Version 1 08/22/2025

Electronic publication
