# Supplementary material for: Single-cell spatial transcriptomics unravels the cellular landscape of abdominal aortic aneurysm
Source: JCI Insight. 2025 Aug 22;10(16):e190534. doi: 10.1172/jci.insight.190534 (PMC12406718; doi:10.1172/jci.insight.190534)
Supplement: Supplemental data [file jciinsight-10-190534-s266.pdf]

SUPPLEMENTAL MATERIALS

For

Single-Cell Spatial Transcriptomics Unravels the Cellular Landscape of Abdominal Aortic Aneurysm

Guizhen Zhao*, Chun-Seok Cho*, Hongyu Liu, Yongha Hwang, Yichen Si, Myungjin Kim, Yongjie Deng, Yang Zhao, Chao Xue, Yanhong Guo, Lin Chang, Dogukan Mizrak, Bo Yang, Hyun Min Kang, Jifeng Zhang, Jun Hee Lee, Y. Eugene Chen

* The authors contributed equally to this article.

Contents:

Supplemental Figures 1 through 8 and Figure Legends.

Supplemental Tables 1 through 4.

Major Resource Table

Supplemental Figures and Legends

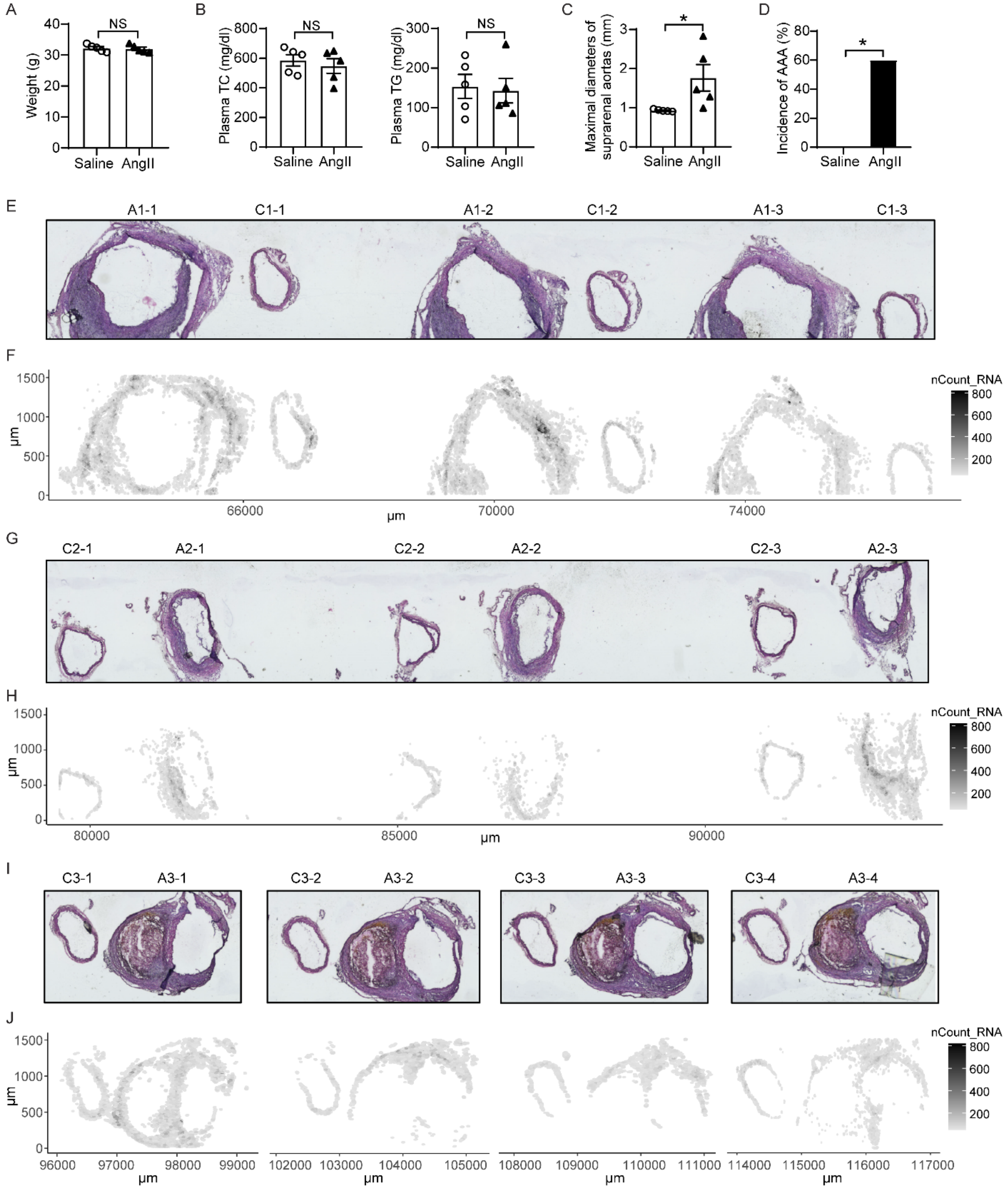

Supplemental Figure 1. AngII infusion induction of abdominal aortic aneurysm in mouse and Seq-Scope analysis of suprarenal abdominal aorta. 16-week-old male

Apoe$^{-/-}$ mice were infused AngII (1,000 ng/kg/min) or saline with minipumps for 4 weeks. n=5/group. **A-B**, Body weight, plasms total cholesterol (TC) and triglycerides (TG) levels after 4 weeks AngII infusion. **C**, Quantification of the maximum external diameters of suprarenal abdominal aortas. **D**, AAA incidence. **E, G** and **I,** Representative hematoxylin and eosin (H&E) staining of mouse suprarenal abdominal aortas from normal controls and AAAs. **F**, **H** and **J**, Hexagons with gene expression information corresponded to the areas under aortic sections in E, G and I were analyzed by spatial transcriptomics (Seq-Scope). Data are represented as mean±SEM. Student's t test for A-C. Chi-squared test for D. NS, no significance. *P<0.05.

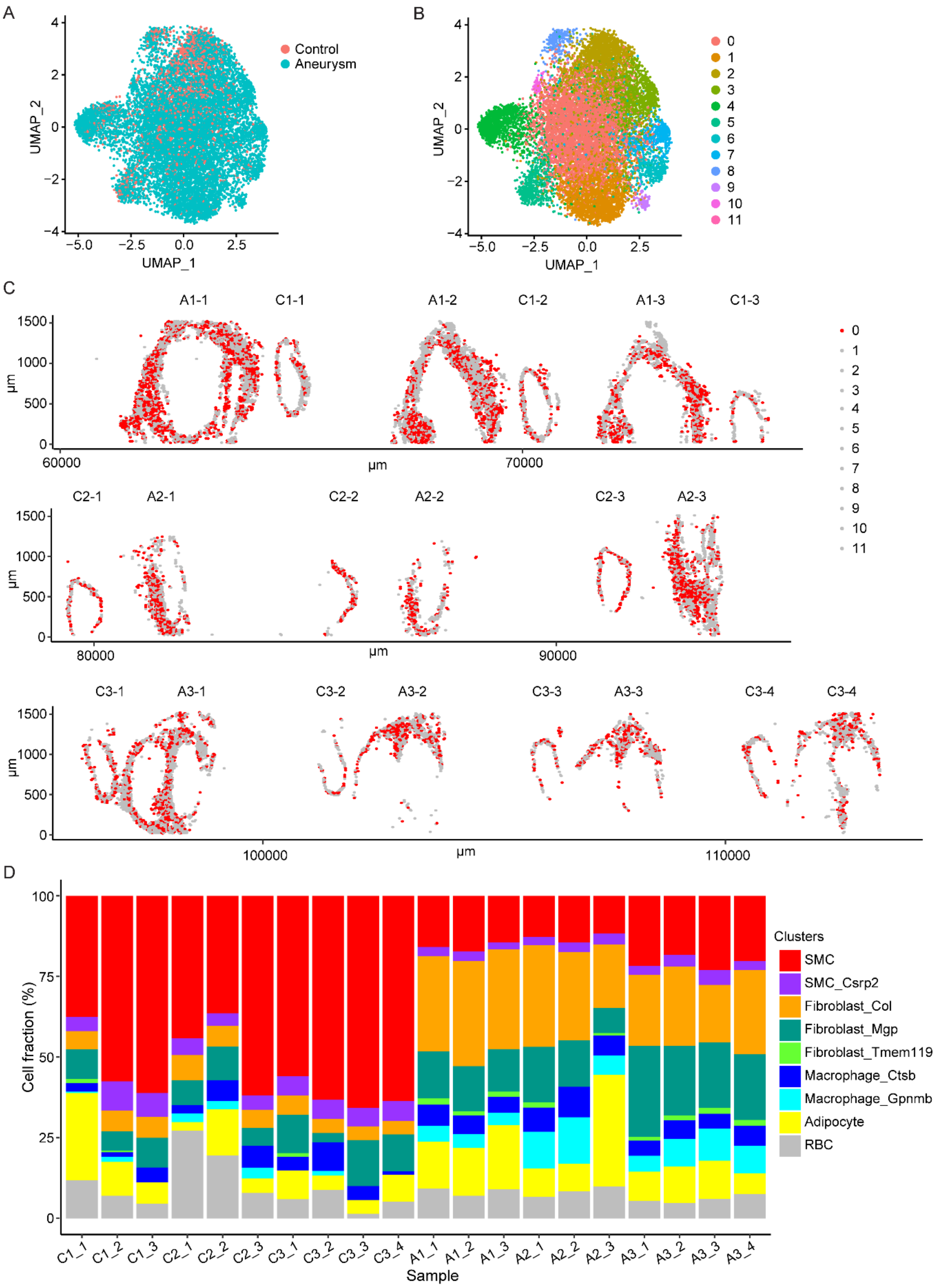
A
UMAP_1
UMAP_2
Control
Aneurysm
B
UMAP_1
UMAP_2
0
1
2
3
4
5
6
7
8
9
10
11
C
A1-1
C1-1
A1-2
C1-2
A1-3
C1-3
C2-1
A2-1
C2-2
A2-2
C2-3
A2-3
C3-1
A3-1
C3-2
A3-2
C3-3
A3-3
C3-4
C3-4
μm
D
Cell fraction (%)
Sample
Clusters
SMC
SMC_Csrp2
Fibroblast_Col
Fibroblast_Mgp
Fibroblast_Tmem119
Macrophage_Ctsb
Macrophage_Gpnmb
Adipocyte
RBC
C1_1
C1_2
C1_3
C2_1
C2_2
C2_3
C3_1
C3_2
C3_3
C3_4
A1_1
A1_2
A1_3
A2_1
A2_2
A2_3
A3_1
A3_2
A3_3
A3_4

Supplemental Figure 2. Spatial transcriptomics analysis of mouse normal and aneurysmal suprarenal abdominal aortas. A, UMAP of all spatial transcriptomics spots from normal control and aneurysm groups, which include 10 sections from 3 normal aortas and 10 sections from 3 aneurysmal aortas. **B**, UMAP visualization of spatial transcriptomics spots based on cell-type clusters. **C**, Spatial plots of indicated cell-type clusters. Cluster 0, red. Cluster 1-11, grey. **D**, Cell cluster (excluding clusters 0, 10 and 11) percentages in each aortic section. SMC, smooth muscle cells. Csrp2, SMC_Csrp2, SMC highly expressing cysteine and glycine-rich protein 2. Fibroblast_Col, fibroblast highly expressing collagen. Fibroblast_Mgp, fibroblast highly expressing matrix Gla protein. Firboblast_Tmem119, fibroblast highly expressing transmembrane protein 119. Macrophage_Ctsb, macrophage highly expressing cathepsin B. Macrophage_Gpnmb, macrophage highly expressing glycoprotein Nmb. RBC, red blood cell. In all spatial plots, the axis units are in μm.

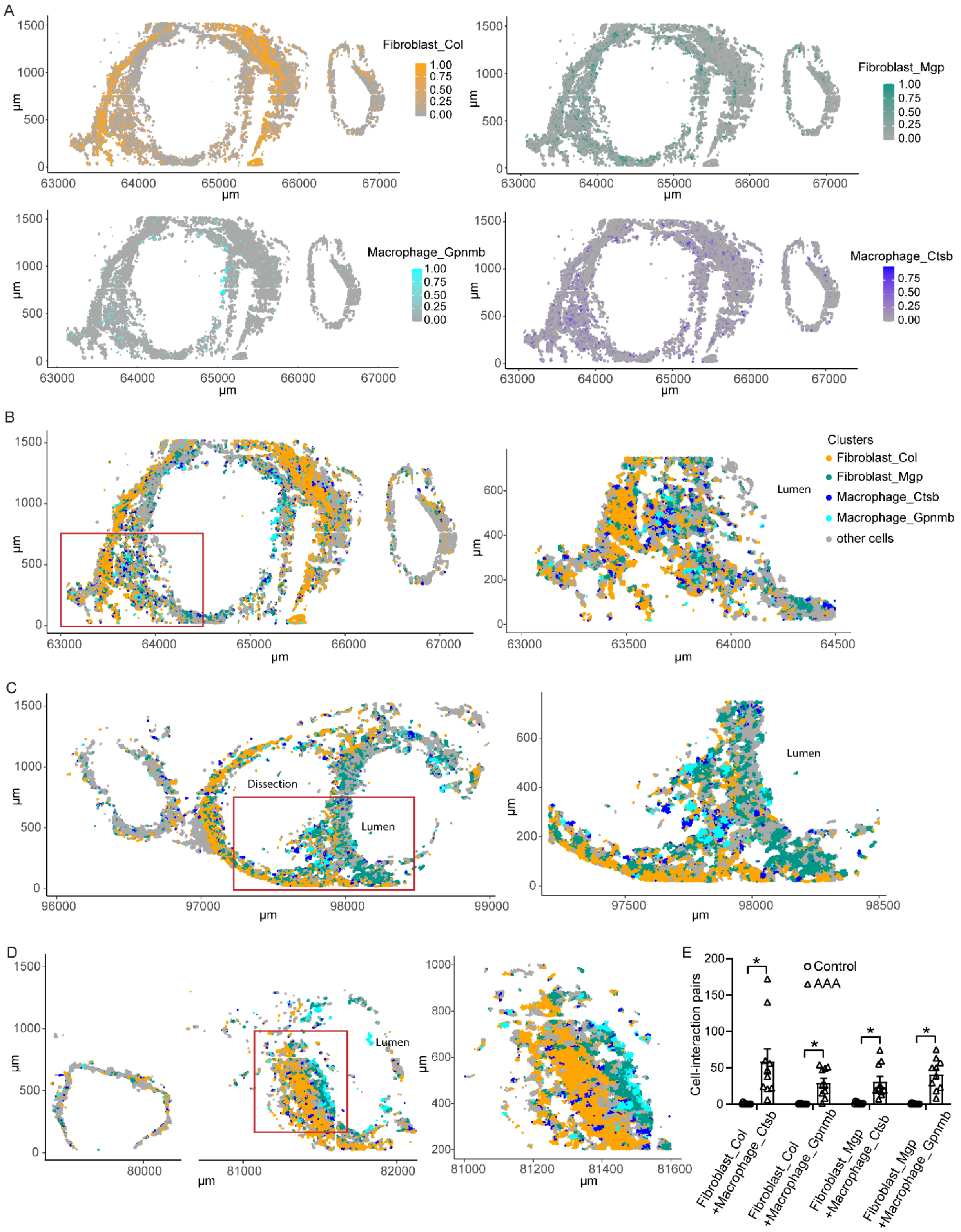
A
Fibroblast_Col
1.00
0.75
0.50
0.25
0.00
Fibroblast_Mgp
Macrophage_Gpnmb
Macrophage_Ctsb
µm
B
Clusters
Fibroblast_Col
Fibroblast_Mgp
Macrophage_Ctsb
Macrophage_Gpnmb
other cells
Lumen
C
Dissection
Lumen
D
Lumen
E
Cell-interaction pairs
Control
AAA
*
Fibroblast_Col +Macrophage_Ctsb
Fibroblast_Col +Macrophage_Gpnmb
Fibroblast_Mgp +Macrophage_Ctsb
Fibroblast_Mgp +Macrophage_Gpnmb

Supplemental Figure 3. Spatial mapping of macrophages and fibroblasts in normal aorta and AAA. **A**, Spatial plot showing the spatial prediction score of Fibroblast_Col, Fibroblast_Mgp, Macrophage_Gpnmb, and Macrophage_Ctsb in the aortic tissues. **B-D**, Spatial plot visualizing locations of Fibroblast_Col, Fibroblast_Mgp, Macrophage_Gpnmb, and Macrophage_Ctsb in the tissue sections. The boxed areas (red boxes) are magnified on the right. In all spatial plots, the axis units are in μm. **E**, Quantification of cell-interaction pairs between Fibroblast_Col, Fibroblast_Mgp and Macrophage_Gpnmb, Macrophage_Ctsb in the tissue sections from normal control and aneurysm groups (10 sections for each group). Cell-cell interaction pairs were quantified using a Euclidean distance threshold of 45 μm. Data are represented as mean±SEM. Student's t test for E. $^*P<0.05$.

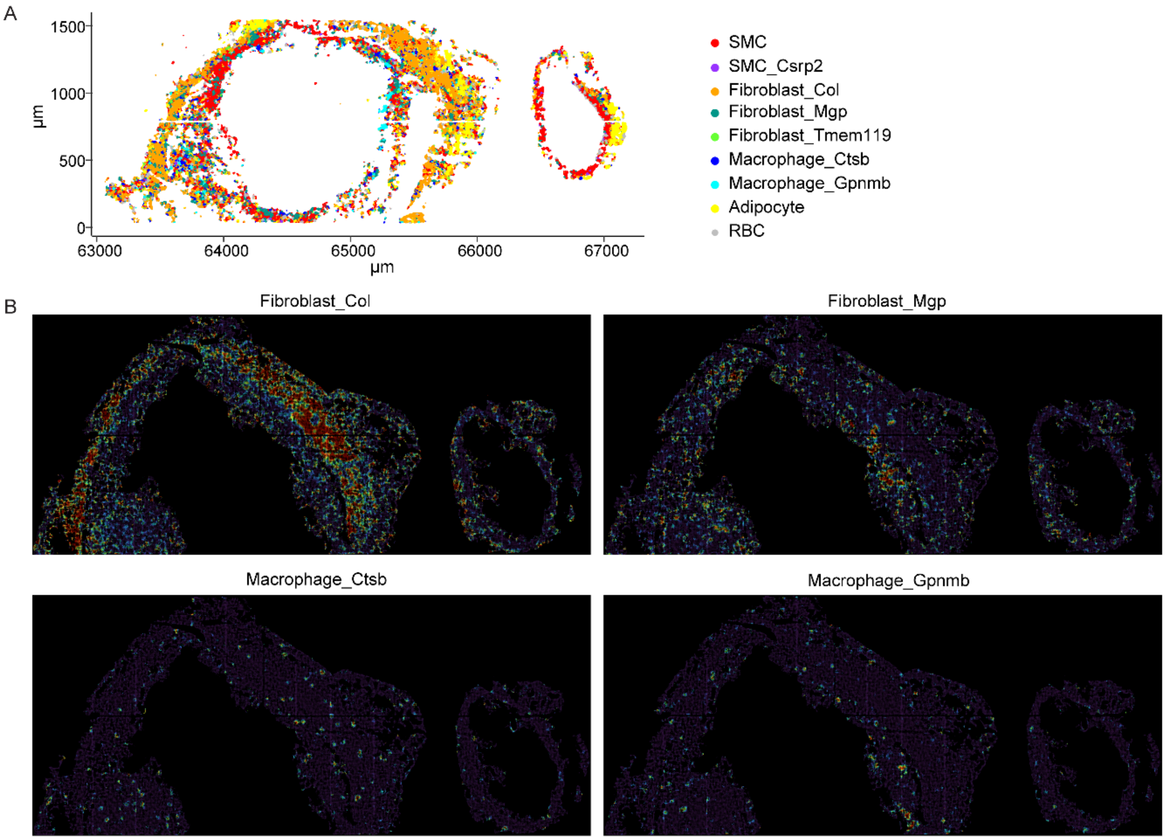

Supplemental Figure 4. Spatial probability distribution of fibroblast and macrophage subpopulations using FICTURE. A, Spatial plots of cell-type clusters identified through Seurat. **B**, Spatial probability distribution of Fibroblast_Col, Fibroblast_Mgp, Macrophage_Ctsb and Macrophage_Gpnmb subpopulations using FICTURE (Factor Inference of Cartographic Transcriptome at Ultra-high Resolution).

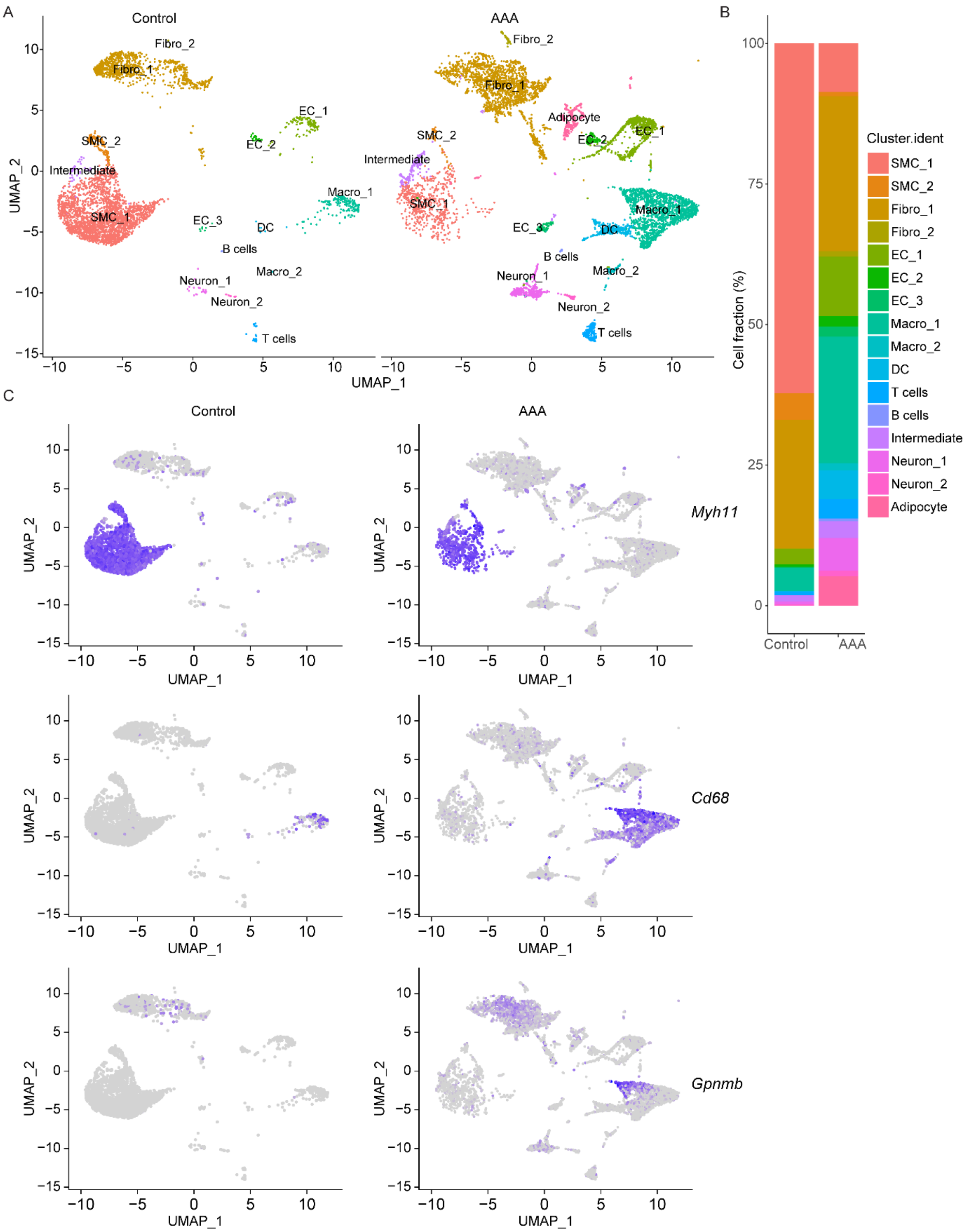
A
Control
AAA
Fibro_2
Fibro_1
SMC_2
Intermediate
SMC_1
EC_1
EC_2
EC_3
DC
Macro_1
Macro_2
B cells
Neuron_1
Neuron_2
T cells
Adipocyte
UMAP_1
UMAP_2
B
Cell fraction (%)
Control
AAA
Cluster.ident
SMC_1
SMC_2
Fibro_1
Fibro_2
EC_1
EC_2
EC_3
Macro_1
Macro_2
DC
T cells
B cells
Intermediate
Neuron_1
Neuron_2
Adipocyte
C
Myh11
Cd68
Gpnmb

Supplemental Figure 5. Single-cell RNA-sequencing analysis of healthy suprarenal abdominal aorta and AngII-induced aneurysmal abdominal aorta. The data were reanalyzed from the datasets GSE193265 and GSE191226. Eight-week-old male ApoE$^{-/-}$ mice were implanted with minipumps to infuse AngII (1,000 ng/kg/min) for 4 weeks. The supernal abdominal aorta from AngII-infused mice or control mice were dissociated into single cells for scRNA-seq. **A**, UMAP plot of cell clusters in abdominal aortas of control and AAA groups. **B**, Cell population percentages in abdominal aortas across control and AAA groups. **C**, Feature plots showing the expression of Myh11, Cd68 and Gpnmb in abdominal aortic cells from control and AAA groups.

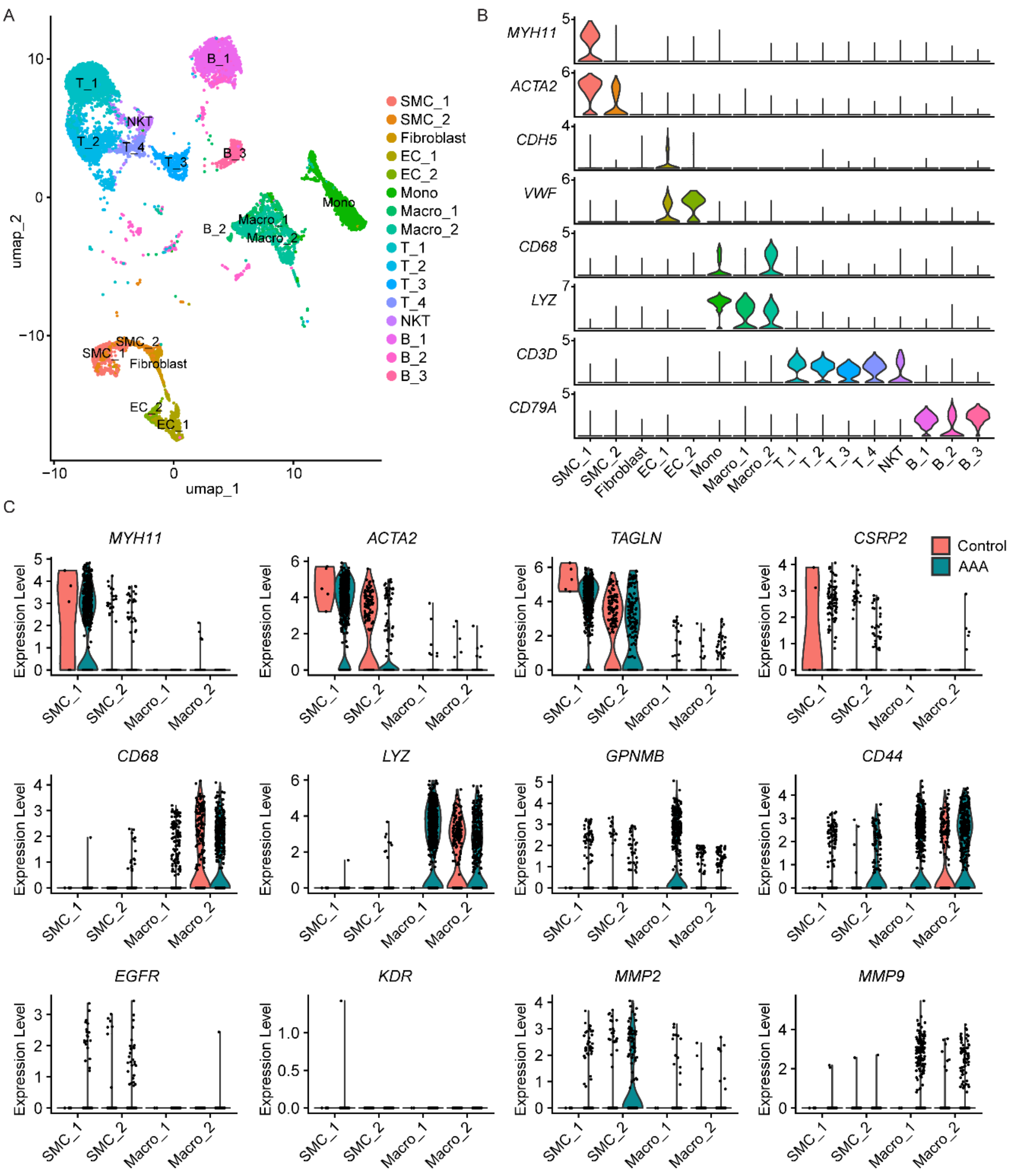

Supplemental Figure 6. Single-cell RNA-sequencing analysis of human abdominal aortic tissues from patients with AAA or control aorta. The data were reanalyzed from the dataset GSE166676 and GSE237230. Fresh human aortic tisse from patients with AAA (4 samples in GSE166676, 4 samples in GSE237230) or nonaneurysmal

control aorta (2 samples in GSE166676). **A**, UMAP plot of cell clusters in human abdominal aortas. **B**, StackedVlnPlot showing the representative marker genes for each cell type. **C**, Violin plot showing the expression of *MYH11*, *ACTA2, TAGLN*, *CSRP2*, *CD68*, *LYZ*, *GPNMB*, *CD44*, *EGFR*, *KDR*, *MMP2* and *MMP9* in SMC and macrophage subpopulations across the control and AAA conditions.

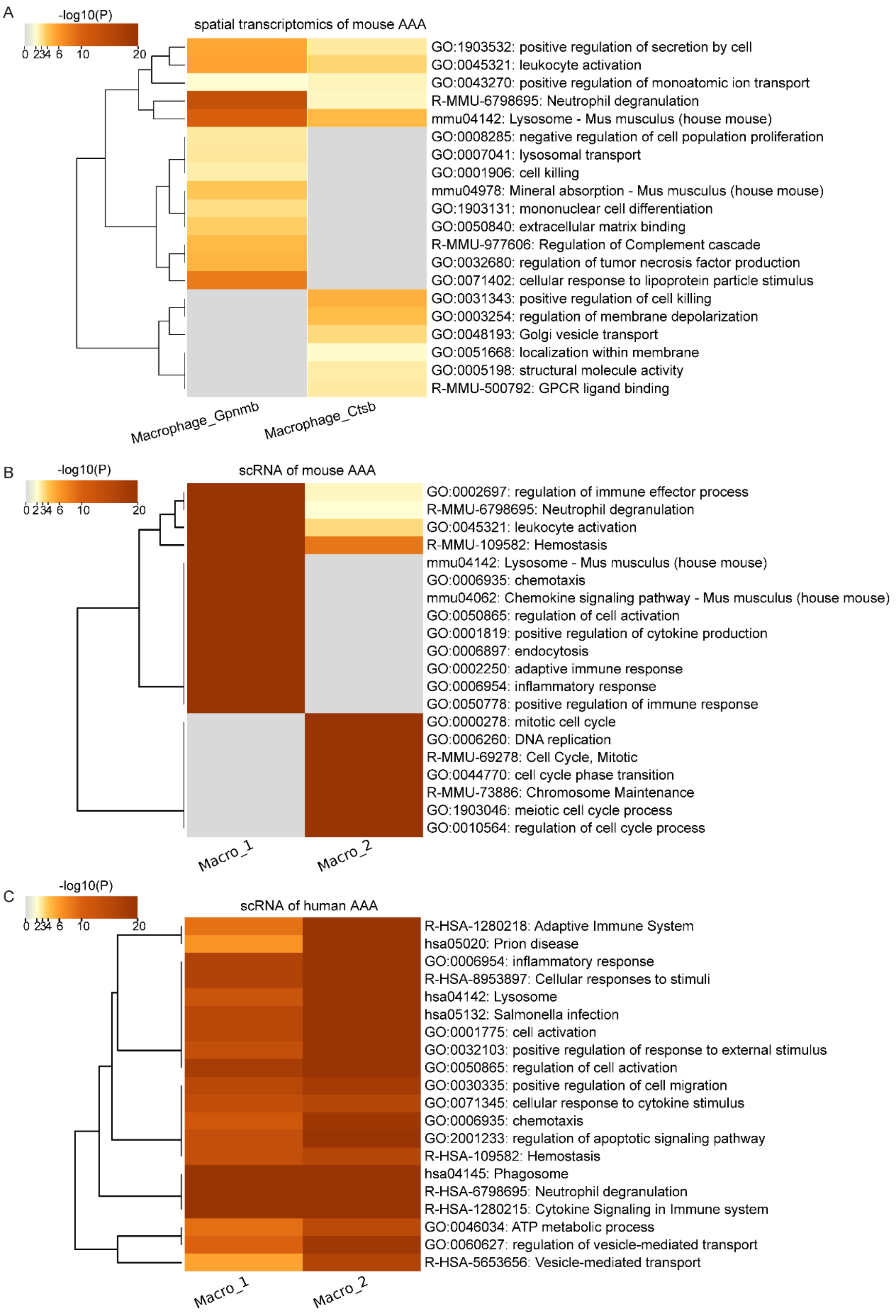
A
-log10(P)
0 2 3 4 6 10 20
spatial transcriptomics of mouse AAA
GO:1903532: positive regulation of secretion by cell
GO:0045321: leukocyte activation
GO:0043270: positive regulation of monoatomic ion transport
R-MMU-6798695: Neutrophil degranulation
mmu04142: Lysosome - Mus musculus (house mouse)
GO:0008285: negative regulation of cell population proliferation
GO:0007041: lysosomal transport
GO:0001906: cell killing
mmu04978: Mineral absorption - Mus musculus (house mouse)
GO:1903131: mononuclear cell differentiation
GO:0050840: extracellular matrix binding
R-MMU-977606: Regulation of Complement cascade
GO:0032680: regulation of tumor necrosis factor production
GO:0071402: cellular response to lipoprotein particle stimulus
GO:0031343: positive regulation of cell killing
GO:0003254: regulation of membrane depolarization
GO:0048193: Golgi vesicle transport
GO:0051668: localization within membrane
GO:0005198: structural molecule activity
R-MMU-500792: GPCR ligand binding
Macrophage_Gpnmb
Macrophage_Ctsb
B
-log10(P)
0 2 3 4 6 10 20
scRNA of mouse AAA
GO:0002697: regulation of immune effector process
R-MMU-6798695: Neutrophil degranulation
GO:0045321: leukocyte activation
R-MMU-109582: Hemostasis
mmu04142: Lysosome - Mus musculus (house mouse)
GO:0006935: chemotaxis
mmu04062: Chemokine signaling pathway - Mus musculus (house mouse)
GO:0050865: regulation of cell activation
GO:0001819: positive regulation of cytokine production
GO:0006897: endocytosis
GO:0002250: adaptive immune response
GO:0006954: inflammatory response
GO:0050778: positive regulation of immune response
GO:0000278: mitotic cell cycle
GO:0006260: DNA replication
R-MMU-69278: Cell Cycle, Mitotic
GO:0044770: cell cycle phase transition
R-MMU-73886: Chromosome Maintenance
GO:1903046: meiotic cell cycle process
GO:0010564: regulation of cell cycle process
Macro_1
Macro_2
C
-log10(P)
0 2 3 4 6 10 20
scRNA of human AAA
R-HSA-1280218: Adaptive Immune System
hsa05020: Prion disease
GO:0006954: inflammatory response
R-HSA-8953897: Cellular responses to stimuli
hsa04142: Lysosome
hsa05132: Salmonella infection
GO:0001775: cell activation
GO:0032103: positive regulation of response to external stimulus
GO:0050865: regulation of cell activation
GO:0030335: positive regulation of cell migration
GO:0071345: cellular response to cytokine stimulus
GO:0006935: chemotaxis
GO:2001233: regulation of apoptotic signaling pathway
R-HSA-109582: Hemostasis
hsa04145: Phagosome
R-HSA-6798695: Neutrophil degranulation
R-HSA-1280215: Cytokine Signaling in Immune system
GO:0046034: ATP metabolic process
GO:0060627: regulation of vesicle-mediated transport
R-HSA-5653656: Vesicle-mediated transport
Macro_1
Macro_2

Supplemental Figure 7. Functional and pathway enrichment analysis of macrophage subpopulations. Heatmap showing the Gene Ontology (GO) and KEGG-terms enrichment of the cluster-specific genes for the macrophage subpopulations in the spatial transcriptomic data (**A**), reanalyzed scRNA-seq mouse aortic data (GSE193265 and GSE191226, **B**) and human aortic data (GSE166676 and GSE237230, C).

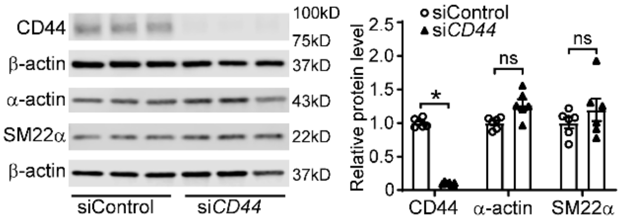

Supplemental Figure 8. CD44 knockdown did not affect the basal expression of SMC marker proteins. Human aortic smooth muscle cells were transfected with nontargeting control siRNA (siControl) or si*CD44*, following by serum-starved for 24h. The protein abundance of smooth muscle α-actin, calponin and SM22α was determined by Western blot. Data are represented as mean±SEM. Student's t test. **P*<0.05. ns, not significant.

Supplemental Table 1. The overall quality of Seq-Scope

Number of Reads	778045214
Reads With Valid Barcodes	0.481664
Sequencing Saturation	0.81113
Q30 Bases in CB+UMI	0.960287
Q30 Bases in RNA read	0.84374
Reads Mapped to Genome: Unique+Multiple	0.916232
Reads Mapped to Genome: Unique	0.73462
Reads Mapped to GeneFull: Unique+Multipe GeneFull	0.142683
Reads Mapped to GeneFull: Unique GeneFull	0.126213

Supplemental Table 2. The differentially expressed markers for all clusters

gene	p_val	avg_log2FC	pct.1	pct.2	p_val_adj	cluster	Annotated cell type
Col1a2	3.05E-199	0.505552	0.491	0.195	4.58E-195	1	
Igfbp4	5.44E-181	0.417270	0.39	0.133	8.17E-177	1	
Fbln1	6.61E-166	0.267426	0.223	0.048	9.91E-162	1	
Col3a1	3.04E-165	0.518699	0.591	0.31	4.57E-161	1	
Cst3	5.71E-163	0.525356	0.535	0.259	8.57E-159	1	
Dcn	1.05E-124	0.265829	0.259	0.08	1.57E-120	1	
Col1a1	4.21E-82	0.256998	0.281	0.119	6.32E-78	1	
C3	1.81E-79	0.270583	0.294	0.13	2.71E-75	1	
Gpx3	3.74E-55	0.164125	0.159	0.059	5.61E-51	1	
Lum	1.66E-35	0.104086	0.119	0.048	2.49E-31	1	
Loxl1	9.54E-28	0.089558	0.101	0.042	1.43E-23	1	
Sparc	2.13E-19	0.122058	0.237	0.155	3.20E-15	1	
Grn	6.11E-16	0.093362	0.141	0.084	9.16E-12	1	
Rps21	1.77E-13	0.087817	0.189	0.127	2.65E-09	1	
Rps19	3.35E-13	0.078896	0.146	0.092	5.03E-09	1	
Tpt1	2.15E-12	0.095272	0.357	0.278	3.22E-08	1	
Rpl37	4.23E-12	0.083434	0.161	0.107	6.34E-08	1	
Rps27a	1.24E-11	0.079982	0.16	0.107	1.86E-07	1	
H2-D1	9.15E-11	0.065377	0.128	0.083	1.37E-06	1	
Rps28	6.98E-10	0.074533	0.168	0.119	1.05E-05	1	
Rps12	1.95E-09	0.070280	0.158	0.111	2.92E-05	1	Fibroblast_Col
Mgp	4.16E-09	0.036372	0.664	0.577	6.24E-05	1	
Lamp1	4.96E-09	0.061073	0.137	0.094	7.44E-05	1	
Ubc	5.75E-09	0.066101	0.131	0.089	8.63E-05	1	
Rps9	1.05E-08	0.065952	0.176	0.128	0.000158	1	
Rps13	1.83E-08	0.066430	0.192	0.143	0.000275	1	
Bgn	2.61E-08	0.079005	0.288	0.23	0.000391	1	
Rpl13	6.21E-08	0.056240	0.158	0.115	0.000931	1	
Selenop	1.72E-07	0.067882	0.21	0.162	0.002580	1	
Rack1	1.76E-07	0.060291	0.151	0.11	0.002642	1	
Rps20	5.97E-07	0.062540	0.173	0.131	0.008951	1	
Rps14	9.16E-07	0.050174	0.114	0.081	0.013738	1	
Rps29	2.74E-06	0.069478	0.271	0.224	0.041119	1	
Rps24	7.54E-06	0.052833	0.193	0.152	0.113126	1	
Serinc3	1.29E-05	0.040663	0.136	0.102	0.193181	1	
Rpl32	1.56E-05	0.046111	0.124	0.092	0.233726	1	
Rps3	2.93E-05	0.040949	0.118	0.088	0.439015	1	
Rpl35a	0.000238	0.037973	0.12	0.093	1	1	
Bsg	0.000292	0.037983	0.115	0.089	1	1	
Rpl13a	0.000343	0.051320	0.24	0.205	1	1	

Rplp1	0.000641	0.046427	0.241	0.207	1	1	
Rpl31	0.000770	0.036812	0.134	0.108	1	1	
Rps15	0.000993	0.036479	0.102	0.08	1	1	
Rps23	0.001188	0.037648	0.153	0.126	1	1	
Rps4x	0.001380	0.033985	0.109	0.087	1	1	
Rps26	0.001910	0.032471	0.117	0.095	1	1	
Anxa5	0.002073	0.028137	0.103	0.082	1	1	
Itm2b	0.005314	0.024463	0.103	0.084	1	1	
Eef2	0.008276	0.039619	0.208	0.183	1	1	
Atp5e	0.008873	0.029657	0.1	0.082	1	1	
Acta2	0	0.711127	0.696	0.276	0	2	SMC
Tagln	1.00E-270	0.518067	0.464	0.133	1.50E-266	2	
Myl9	2.48E-223	0.492624	0.438	0.139	3.72E-219	2	
Cpe	2.46E-116	0.326293	0.316	0.116	3.69E-112	2	
Ptgis	4.11E-72	0.179505	0.147	0.042	6.16E-68	2	
Flna	8.45E-69	0.236445	0.236	0.097	1.27E-64	2	
Col18a1	5.13E-51	0.120147	0.118	0.037	7.70E-47	2	
Tpm2	1.61E-49	0.140590	0.144	0.052	2.41E-45	2	
Ccn2	2.13E-49	0.145494	0.144	0.052	3.20E-45	2	
Myl6	7.61E-32	0.184169	0.284	0.173	1.14E-27	2	
Csrp1	4.65E-31	0.120677	0.125	0.054	6.97E-27	2	
Mfge8	1.08E-29	0.132616	0.132	0.06	1.62E-25	2	
Serpine2	2.41E-24	0.092178	0.107	0.048	3.61E-20	2	
Fn1	5.43E-22	0.162890	0.296	0.2	8.15E-18	2	
Dstn	5.02E-20	0.109785	0.152	0.085	7.53E-16	2	
Bgn	5.27E-15	0.135319	0.308	0.228	7.90E-11	2	
Sod3	7.22E-15	0.073578	0.1	0.053	1.08E-10	2	
S100a6	4.92E-12	0.086120	0.199	0.137	7.39E-08	2	
Igfbp7	9.87E-12	0.068106	0.114	0.069	1.48E-07	2	
Tmsb4x	2.09E-11	0.096161	0.237	0.173	3.14E-07	2	
Lgals1	8.03E-09	0.070349	0.115	0.075	0.000120	2	
Cavin1	9.08E-09	0.082661	0.128	0.087	0.000136	2	
Actb	1.91E-07	0.105085	0.478	0.423	0.002864	2	
Clu	2.09E-07	0.057032	0.112	0.076	0.003129	2	
Hspa8	0.00142254	0.047402	0.14	0.114	1	2	
Mgp	0	1.195987	0.919	0.555	0	3	Fibroblast_Mgp
Eln	1.92E-191	0.437159	0.326	0.072	2.88E-187	3	
Fn1	8.69E-61	0.329205	0.385	0.195	1.30E-56	3	
Tm4sf1	7.18E-31	0.116639	0.101	0.034	1.08E-26	3	
Ccn2	1.65E-19	0.099416	0.124	0.058	2.48E-15	3	
Cpe	5.28E-17	0.124323	0.222	0.135	7.93E-13	3	
Bgn	1.48E-16	0.173185	0.328	0.229	2.22E-12	3	
Ltbp2	1.59E-13	0.084004	0.109	0.057	2.38E-09	3	
Lgals1	2.09E-06	0.050007	0.115	0.077	0.031396	3	

Col1a1	2.38E-06	0.082714	0.186	0.138	0.035668	3	
Myl6	1.51E-05	0.075171	0.232	0.183	0.226630	3	
Psap	4.40E-05	0.052505	0.26	0.208	0.659637	3	
Actb	5.41E-05	0.081546	0.483	0.425	0.812210	3	
Igfbp7	0.000336	0.036075	0.1	0.072	1	3	
Acta2	0.000449	0.040646	0.382	0.328	1	3	
S100a6	0.000555	0.061759	0.178	0.143	1	3	
Sparc	0.001665	0.054078	0.198	0.164	1	3	
Cst3	0.002838	0.059054	0.334	0.296	1	3	
Tagln	0.003225	0.037334	0.209	0.175	1	3	
Fstl1	0.003721	0.038010	0.105	0.081	1	3	
Dstn	0.008491	0.032778	0.115	0.092	1	3	
Cfd	0.000000	1.469763	0.898	0.139	0	4	Adipose
Hp	2.08E-235	0.536368	0.406	0.091	3.11E-231	4	
Fabp4	4.57E-220	0.427232	0.325	0.061	6.86E-216	4	
Ifi27l2a	3.31E-130	0.232514	0.157	0.022	4.97E-126	4	
Car3	8.60E-112	0.245458	0.186	0.037	1.29E-107	4	
Lpl	3.04E-110	0.265555	0.209	0.047	4.56E-106	4	
Glul	9.93E-63	0.181558	0.16	0.044	1.49E-58	4	
Dbi	5.17E-58	0.232586	0.236	0.09	7.76E-54	4	
Scd1	3.92E-57	0.306435	0.366	0.182	5.88E-53	4	
Cyp2e1	1.85E-38	0.135311	0.122	0.039	2.78E-34	4	
Cox6a1	1.10E-26	0.122913	0.134	0.056	1.64E-22	4	
Cdo1	8.86E-25	0.110677	0.125	0.052	1.33E-20	4	
C4b	8.66E-19	0.099726	0.11	0.049	1.30E-14	4	
Cox8a	1.75E-15	0.109684	0.15	0.083	2.63E-11	4	
Scp2	1.07E-08	0.086017	0.164	0.109	0.000160	4	
Gpx3	5.61E-08	0.061996	0.113	0.07	0.000842	4	
Atp5g3	7.89E-08	0.076758	0.13	0.085	0.001184	4	
H2-D1	1.34E-06	0.069358	0.127	0.086	0.020040	4	
Cox4i1	1.79E-06	0.057016	0.107	0.07	0.026917	4	
Rps28	9.09E-06	0.059511	0.167	0.122	0.136324	4	
C3	1.24E-05	0.075282	0.197	0.15	0.186262	4	
H2-K1	4.05E-05	0.057888	0.135	0.098	0.608271	4	
Mgst1	4.33E-05	0.059257	0.106	0.073	0.649680	4	
Atp5e	0.000797	0.048148	0.11	0.082	1	4	
B2m	0.001293	0.042868	0.134	0.103	1	4	
Atp5a1	0.001727	0.052299	0.128	0.101	1	4	
Chchd2	0.003322	0.056636	0.166	0.137	1	4	
Gpx1	0.009413	0.028515	0.118	0.095	1	4	
Hba-a2	0	0.893265	0.59	0.053	0	5	RBC
Hbb-bs	0	0.596711	0.399	0.018	0	5	
Hbb-bt	2.36E-285	0.224316	0.138	0.002	3.55E-281	5	
Ctsb	0	1.148723	0.916	0.053	0	6	Macrophage_Ctsb

Lamp1	2.68E-07	0.113074	0.171	0.098	0.004014	6	
Psap	0.000147	0.127768	0.282	0.21	1	6	
Ltbp2	0.000724	0.047561	0.1	0.06	1	6	
Rpl34	0.002838	0.059558	0.115	0.077	1	6	
Rpl37	0.007154	0.049507	0.155	0.114	1	6	
Lyz2	0.007804	0.042549	0.102	0.069	1	6	
Gpnmb	0	0.800627	0.569	0.041	0	7	
Cd5l	4.01E-208	0.335780	0.218	0.012	6.02E-204	7	
Ctsd	4.70E-129	0.723536	0.486	0.115	7.05E-125	7	
Psap	1.74E-74	0.667877	0.53	0.202	2.61E-70	7	
Lyz2	2.39E-56	0.313845	0.259	0.064	3.59E-52	7	
Ctsb	2.13E-32	0.232991	0.236	0.077	3.20E-28	7	
Ctsl	7.32E-32	0.230012	0.183	0.052	1.10E-27	7	
Lgals3	9.70E-28	0.127856	0.12	0.028	1.46E-23	7	
Ctss	2.14E-27	0.141128	0.118	0.027	3.21E-23	7	
Ftl1	5.41E-23	0.158043	0.144	0.043	8.11E-19	7	Macrophage_Gpnmb
Atp6v0c	8.72E-14	0.140617	0.157	0.066	1.31E-09	7	
C1qc	5.56E-08	0.083995	0.104	0.047	0.000835	7	
Actb	9.14E-08	0.194078	0.539	0.427	0.001371	7	
Lpl	2.77E-07	0.088255	0.12	0.06	0.004158	7	
Fth1	5.57E-07	0.154109	0.236	0.15	0.008364	7	
Mfge8	5.15E-05	0.084246	0.118	0.068	0.773394	7	
Eln	0.001702	0.056486	0.141	0.095	1	7	
Clu	0.003578	0.075733	0.118	0.08	1	7	
Prdx1	0.004731	0.054021	0.104	0.069	1	7	
Lamp1	0.008037	0.073890	0.137	0.099	1	7	
Csrp2	0	1.129600	0.935	0.022	0	8	
Acta2	1.15E-15	0.384232	0.418	0.208	1.73E-11	8	SMC_Csrp2
Rbp4	0.001208	0.189439	0.169	0.054	0.001525	8	
Tmem119	0	1.144419	0.867	0.004	0	9	
Cst3	5.22E-04	0.542094	0.419	0.125	1.00E+00	9	
Rps29	1.03E-02	0.251539	0.39	0.15	1.00E+00	9	
Eef1a1	2.69E-02	0.332823	0.4	0.225	1.00E+00	9	Fibroblast_Tmem119
Mgp	3.59E-11	0.601679	0.648	0.365	5.38E-07	9	
Col3a1	9.25E-07	0.319955	0.486	0.276	1.39E-02	9	
Col1a2	6.00E-05	0.259455	0.286	0.144	9.00E-01	9	
Rpl28	0	1.342801	0.904	0.03	0	10	Unknown
Ddx17	2.77E-06	0.253954	0.123	0.011	4.16E-02	10	
Ttn	0	0.937618	0.7	0.005	0	11	
Tmcc1	1.44E-91	0.345019	0.225	0.004	2.16E-87	11	

Supplemental Table 3. The differentially expressed markerd for all cell clusters across two conditions

gene	p_val	avg_log2FC	pct.1	pct.2	p_val_adj	cluster	group
Acta2	1.15E-57	0.435266736	0.592	0.373	1.73E-53	SMC	Normal
Myl9	1.45E-28	0.256125267	0.348	0.204	2.17E-24	SMC	Normal
Tagln	4.53E-16	0.180662304	0.312	0.205	6.80E-12	SMC	Normal
Myl6	4.18E-12	0.137594252	0.277	0.189	6.27E-08	SMC	Normal
Ptgis	5.29E-10	0.092291694	0.117	0.064	7.94E-06	SMC	Normal
Cpe	7.26E-09	0.124130489	0.222	0.154	0.000108863	SMC	Normal
Flna	1.34E-08	0.116511459	0.189	0.127	0.000200652	SMC	Normal
Tpm2	1.67E-05	0.056199557	0.113	0.075	0.249856058	SMC	Normal
Csrp1	5.03E-05	0.054796498	0.103	0.068	0.754363384	SMC	Normal
Dstn	0.000487656	0.064815079	0.131	0.097	1	SMC	Normal
Bc1	1.03E-08	0.087727299	0.112	0.056	0.000154757	SMC	AAA
Col3a1	3.58E-08	0.11945377	0.324	0.245	0.000536707	SMC	AAA
Rps24	3.54E-06	0.085730098	0.168	0.114	0.053072655	SMC	AAA
Rplp1	1.25E-05	0.086314252	0.216	0.159	0.187485798	SMC	AAA
Igfbp4	1.67E-05	0.06891686	0.141	0.093	0.251098582	SMC	AAA
Mptx1	3.01E-05	0.088528539	0.424	0.36	0.451217766	SMC	AAA
Rpl13a	3.13E-05	0.089895002	0.219	0.167	0.46973457	SMC	AAA
Col1a1	3.15E-05	0.062793657	0.116	0.074	0.473279493	SMC	AAA
C3	8.05E-05	0.066397556	0.131	0.089	1	SMC	AAA
Actb	0.000316086	0.080457752	0.453	0.397	1	SMC	AAA
Rps20	0.000439718	0.050518676	0.133	0.095	1	SMC	AAA
Ctsd	0.000931295	0.051565575	0.11	0.078	1	SMC	AAA
Rps9	0.002198978	0.051489789	0.138	0.105	1	SMC	AAA
Rps28	0.003082831	0.050915377	0.116	0.086	1	SMC	AAA
Apoe	0.003535891	0.059748784	0.387	0.343	1	SMC	AAA
Serinc3	0.004197199	0.040419355	0.104	0.076	1	SMC	AAA

Rpl23	0.005304712	0.044020064	0.133	0.103	1	SMC	AAA
Atp5a1	0.009926521	0.032951493	0.11	0.084	1	SMC	AAA

Acta2	0.000274679	0.371736324	0.563	0.363	1	SMC_Csrp2	Normal
Flna	0.002655554	0.210087992	0.268	0.118	1	SMC_Csrp2	Normal
Csrp2	0.003088378	0.17445883	0.986	0.922	1	SMC_Csrp2	Normal
Serf2	0.007048934	0.142830152	0.155	0.054	1	SMC_Csrp2	Normal

Mpc2	1.57E-06	0.125428761	0.116	0.022	0.023489085	Fibroblast_Col	Normal
Col3a1	7.12E-05	0.41033719	0.606	0.391	1	Fibroblast_Col	AAA
C3	0.002142391	0.294526498	0.294	0.13	1	Fibroblast_Col	AAA
Gpx3	0.003147729	0.21616518	0.159	0.029	1	Fibroblast_Col	AAA
Actb	0.004808424	0.246988497	0.424	0.246	1	Fibroblast_Col	AAA
Fbln1	0.005243444	0.220149349	0.227	0.087	1	Fibroblast_Col	AAA
Mup20	0.007303023	0.19046607	0.243	0.101	1	Fibroblast_Col	AAA
Rpl31	0.008720601	0.159103817	0.139	0.029	1	Fibroblast_Col	AAA
Col1a1	0.008956186	0.180292639	0.292	0.145	1	Fibroblast_Col	AAA
Rpl27a	0.00517867	0.151616881	0.102	0	1	Fibroblast_Col	AAA
H2-D1	0.009550203	0.145153379	0.114	0.014	1	Fibroblast_Col	AAA
Lum	0.007135552	0.139985472	0.123	0.014	1	Fibroblast_Col	AAA

Mylk	1.98E-05	0.156949946	0.139	0.044	0.296898285	Fibroblast_Mgp	Normal
Tns1	0.000709179	0.112399374	0.102	0.035	1	Fibroblast_Mgp	Normal
Cyp3a11	0.000285335	0.095661737	0.105	0.033	1	Fibroblast_Mgp	Normal
Mgp	3.29E-05	0.313225643	0.926	0.852	0.493043365	Fibroblast_Mgp	AAA
Fn1	0.000844504	0.261322508	0.398	0.238	1	Fibroblast_Mgp	AAA
Ctsd	0.002208425	0.16615981	0.158	0.048	1	Fibroblast_Mgp	AAA
Actb	0.002302947	0.213951908	0.496	0.343	1	Fibroblast_Mgp	AAA
Col1a2	0.004275564	0.197103517	0.256	0.133	1	Fibroblast_Mgp	AAA

Col3a1	0.007666426	0.198512694	0.369	0.248	1	Fibroblast_Mgp	AAA

Ndufs6	1.57E-09	0.570462931	0.5	0.01	2.35E-05	Fibroblast_Tmem119	Normal
Tcp1	9.29E-09	0.415037499	0.333	0	0.00013937	Fibroblast_Tmem119	Normal
D130017N08Rik	5.80E-05	0.415037499	0.167	0	0.869547578	Fibroblast_Tmem119	Normal
Liph	5.80E-05	0.415037499	0.167	0	0.869547578	Fibroblast_Tmem119	Normal
Tnfrsf23	5.80E-05	0.415037499	0.167	0	0.869547578	Fibroblast_Tmem119	Normal
Tnrc6c	5.80E-05	0.415037499	0.167	0	0.869547578	Fibroblast_Tmem119	Normal
Vmn2r50	5.80E-05	0.415037499	0.167	0	0.869547578	Fibroblast_Tmem119	Normal
AI427809	5.80E-05	0.222392421	0.167	0	0.869547578	Fibroblast_Tmem119	Normal
Abcg2	5.80E-05	0.222392421	0.167	0	0.869547578	Fibroblast_Tmem119	Normal
Adra2a	5.80E-05	0.222392421	0.167	0	0.869547578	Fibroblast_Tmem119	Normal
Aldh18a1	5.80E-05	0.222392421	0.167	0	0.869547578	Fibroblast_Tmem119	Normal
Anapc5	5.80E-05	0.222392421	0.167	0	0.869547578	Fibroblast_Tmem119	Normal
Aox1	5.80E-05	0.222392421	0.167	0	0.869547578	Fibroblast_Tmem119	Normal
Apaf1	5.80E-05	0.222392421	0.167	0	0.869547578	Fibroblast_Tmem119	Normal
Apc	5.80E-05	0.222392421	0.167	0	0.869547578	Fibroblast_Tmem119	Normal
Appl1	5.80E-05	0.222392421	0.167	0	0.869547578	Fibroblast_Tmem119	Normal
Armc1	5.80E-05	0.222392421	0.167	0	0.869547578	Fibroblast_Tmem119	Normal
Ascc3	5.80E-05	0.222392421	0.167	0	0.869547578	Fibroblast_Tmem119	Normal
Ate1	5.80E-05	0.222392421	0.167	0	0.869547578	Fibroblast_Tmem119	Normal
Aup1	5.80E-05	0.222392421	0.167	0	0.869547578	Fibroblast_Tmem119	Normal
Avpr1a	5.80E-05	0.222392421	0.167	0	0.869547578	Fibroblast_Tmem119	Normal
B130024G19Rik	5.80E-05	0.222392421	0.167	0	0.869547578	Fibroblast_Tmem119	Normal
Birc3	5.80E-05	0.222392421	0.167	0	0.869547578	Fibroblast_Tmem119	Normal
Boc	5.80E-05	0.222392421	0.167	0	0.869547578	Fibroblast_Tmem119	Normal
C1qbp	5.80E-05	0.222392421	0.167	0	0.869547578	Fibroblast_Tmem119	Normal
Cabin1	5.80E-05	0.222392421	0.167	0	0.869547578	Fibroblast_Tmem119	Normal

Calm2	5.80E-05	0.222392421	0.167	0	0.869547578	Fibroblast_Tmem119	Normal
Camsap2	5.80E-05	0.222392421	0.167	0	0.869547578	Fibroblast_Tmem119	Normal
Cd151	5.80E-05	0.222392421	0.167	0	0.869547578	Fibroblast_Tmem119	Normal
Cdc14b	5.80E-05	0.222392421	0.167	0	0.869547578	Fibroblast_Tmem119	Normal
Cdc5l	5.80E-05	0.222392421	0.167	0	0.869547578	Fibroblast_Tmem119	Normal
Cdh18	5.80E-05	0.222392421	0.167	0	0.869547578	Fibroblast_Tmem119	Normal
Cdh2	5.80E-05	0.222392421	0.167	0	0.869547578	Fibroblast_Tmem119	Normal
Ciart	5.80E-05	0.222392421	0.167	0	0.869547578	Fibroblast_Tmem119	Normal
Clptm1	5.80E-05	0.222392421	0.167	0	0.869547578	Fibroblast_Tmem119	Normal
Col5a2	5.80E-05	0.222392421	0.167	0	0.869547578	Fibroblast_Tmem119	Normal
Ctdnep1	5.80E-05	0.222392421	0.167	0	0.869547578	Fibroblast_Tmem119	Normal
Dazap1	5.80E-05	0.222392421	0.167	0	0.869547578	Fibroblast_Tmem119	Normal
Ddx54	5.80E-05	0.222392421	0.167	0	0.869547578	Fibroblast_Tmem119	Normal
Desi2	5.80E-05	0.222392421	0.167	0	0.869547578	Fibroblast_Tmem119	Normal
Dhx29	5.80E-05	0.222392421	0.167	0	0.869547578	Fibroblast_Tmem119	Normal
Dhx33	5.80E-05	0.222392421	0.167	0	0.869547578	Fibroblast_Tmem119	Normal
Dld	5.80E-05	0.222392421	0.167	0	0.869547578	Fibroblast_Tmem119	Normal
Ebp	5.80E-05	0.222392421	0.167	0	0.869547578	Fibroblast_Tmem119	Normal
Enah	5.80E-05	0.222392421	0.167	0	0.869547578	Fibroblast_Tmem119	Normal
Evi5	5.80E-05	0.222392421	0.167	0	0.869547578	Fibroblast_Tmem119	Normal
Faf2	5.80E-05	0.222392421	0.167	0	0.869547578	Fibroblast_Tmem119	Normal
Fam193a	5.80E-05	0.222392421	0.167	0	0.869547578	Fibroblast_Tmem119	Normal
Fam222b	5.80E-05	0.222392421	0.167	0	0.869547578	Fibroblast_Tmem119	Normal
Fbxw5	5.80E-05	0.222392421	0.167	0	0.869547578	Fibroblast_Tmem119	Normal
Fibin	5.80E-05	0.222392421	0.167	0	0.869547578	Fibroblast_Tmem119	Normal
Foxk2	5.80E-05	0.222392421	0.167	0	0.869547578	Fibroblast_Tmem119	Normal
Foxn3	5.80E-05	0.222392421	0.167	0	0.869547578	Fibroblast_Tmem119	Normal

Fundc2	5.80E-05	0.222392421	0.167	0	0.869547578	Fibroblast_Tmem119	Normal
Glyr1	5.80E-05	0.222392421	0.167	0	0.869547578	Fibroblast_Tmem119	Normal
Gria2	5.80E-05	0.222392421	0.167	0	0.869547578	Fibroblast_Tmem119	Normal
Grpel1	5.80E-05	0.222392421	0.167	0	0.869547578	Fibroblast_Tmem119	Normal
Hao1	5.80E-05	0.222392421	0.167	0	0.869547578	Fibroblast_Tmem119	Normal
Hdx	5.80E-05	0.222392421	0.167	0	0.869547578	Fibroblast_Tmem119	Normal
Hes6	5.80E-05	0.222392421	0.167	0	0.869547578	Fibroblast_Tmem119	Normal
Hoxa2	5.80E-05	0.222392421	0.167	0	0.869547578	Fibroblast_Tmem119	Normal
Hpn	5.80E-05	0.222392421	0.167	0	0.869547578	Fibroblast_Tmem119	Normal
Hsd17b4	5.80E-05	0.222392421	0.167	0	0.869547578	Fibroblast_Tmem119	Normal
Hsd17b6	5.80E-05	0.222392421	0.167	0	0.869547578	Fibroblast_Tmem119	Normal
Itga8	5.80E-05	0.222392421	0.167	0	0.869547578	Fibroblast_Tmem119	Normal
Kit	5.80E-05	0.222392421	0.167	0	0.869547578	Fibroblast_Tmem119	Normal
Lamb2	5.80E-05	0.222392421	0.167	0	0.869547578	Fibroblast_Tmem119	Normal
Lcat	5.80E-05	0.222392421	0.167	0	0.869547578	Fibroblast_Tmem119	Normal
Leng8	5.80E-05	0.222392421	0.167	0	0.869547578	Fibroblast_Tmem119	Normal
Lratd2	5.80E-05	0.222392421	0.167	0	0.869547578	Fibroblast_Tmem119	Normal
Lsm2	5.80E-05	0.222392421	0.167	0	0.869547578	Fibroblast_Tmem119	Normal
Mettl9	5.80E-05	0.222392421	0.167	0	0.869547578	Fibroblast_Tmem119	Normal
Mfsd5	5.80E-05	0.222392421	0.167	0	0.869547578	Fibroblast_Tmem119	Normal
Mrpl12	5.80E-05	0.222392421	0.167	0	0.869547578	Fibroblast_Tmem119	Normal
Mrpl48	5.80E-05	0.222392421	0.167	0	0.869547578	Fibroblast_Tmem119	Normal
Myom1	5.80E-05	0.222392421	0.167	0	0.869547578	Fibroblast_Tmem119	Normal
Mysm1	5.80E-05	0.222392421	0.167	0	0.869547578	Fibroblast_Tmem119	Normal
Oard1	5.80E-05	0.222392421	0.167	0	0.869547578	Fibroblast_Tmem119	Normal
Ociad1	5.80E-05	0.222392421	0.167	0	0.869547578	Fibroblast_Tmem119	Normal
Olfr1238	5.80E-05	0.222392421	0.167	0	0.869547578	Fibroblast_Tmem119	Normal

Otud4	5.80E-05	0.222392421	0.167	0	0.869547578	Fibroblast_Tmem119	Normal
P4hb	5.80E-05	0.222392421	0.167	0	0.869547578	Fibroblast_Tmem119	Normal
Pafah2	5.80E-05	0.222392421	0.167	0	0.869547578	Fibroblast_Tmem119	Normal
Pcyt2	5.80E-05	0.222392421	0.167	0	0.869547578	Fibroblast_Tmem119	Normal
Pdia6	5.80E-05	0.222392421	0.167	0	0.869547578	Fibroblast_Tmem119	Normal
Pgpep1	5.80E-05	0.222392421	0.167	0	0.869547578	Fibroblast_Tmem119	Normal
Pim3	5.80E-05	0.222392421	0.167	0	0.869547578	Fibroblast_Tmem119	Normal
Plcb3	5.80E-05	0.222392421	0.167	0	0.869547578	Fibroblast_Tmem119	Normal
Plxdc2	5.80E-05	0.222392421	0.167	0	0.869547578	Fibroblast_Tmem119	Normal
Pot1a	5.80E-05	0.222392421	0.167	0	0.869547578	Fibroblast_Tmem119	Normal
Prkag2	5.80E-05	0.222392421	0.167	0	0.869547578	Fibroblast_Tmem119	Normal
Prxl2c	5.80E-05	0.222392421	0.167	0	0.869547578	Fibroblast_Tmem119	Normal
Purb	5.80E-05	0.222392421	0.167	0	0.869547578	Fibroblast_Tmem119	Normal
Rab22a	5.80E-05	0.222392421	0.167	0	0.869547578	Fibroblast_Tmem119	Normal
Rabl2	5.80E-05	0.222392421	0.167	0	0.869547578	Fibroblast_Tmem119	Normal
Rapgef1	5.80E-05	0.222392421	0.167	0	0.869547578	Fibroblast_Tmem119	Normal
Rev1	5.80E-05	0.222392421	0.167	0	0.869547578	Fibroblast_Tmem119	Normal
Rfx7	5.80E-05	0.222392421	0.167	0	0.869547578	Fibroblast_Tmem119	Normal
Rhog	5.80E-05	0.222392421	0.167	0	0.869547578	Fibroblast_Tmem119	Normal
Riok3	5.80E-05	0.222392421	0.167	0	0.869547578	Fibroblast_Tmem119	Normal
Rnf213	5.80E-05	0.222392421	0.167	0	0.869547578	Fibroblast_Tmem119	Normal
Rpgrip1l	5.80E-05	0.222392421	0.167	0	0.869547578	Fibroblast_Tmem119	Normal
Rrp15	5.80E-05	0.222392421	0.167	0	0.869547578	Fibroblast_Tmem119	Normal
Sccpdh	5.80E-05	0.222392421	0.167	0	0.869547578	Fibroblast_Tmem119	Normal
Senp7	5.80E-05	0.222392421	0.167	0	0.869547578	Fibroblast_Tmem119	Normal
Serpina10	5.80E-05	0.222392421	0.167	0	0.869547578	Fibroblast_Tmem119	Normal
Sh2d3c	5.80E-05	0.222392421	0.167	0	0.869547578	Fibroblast_Tmem119	Normal

Slc16a1	5.80E-05	0.222392421	0.167	0	0.869547578	Fibroblast_Tmem119	Normal
Slc16a7	5.80E-05	0.222392421	0.167	0	0.869547578	Fibroblast_Tmem119	Normal
Slc35e4	5.80E-05	0.222392421	0.167	0	0.869547578	Fibroblast_Tmem119	Normal
Smim4	5.80E-05	0.222392421	0.167	0	0.869547578	Fibroblast_Tmem119	Normal
Smpdl3a	5.80E-05	0.222392421	0.167	0	0.869547578	Fibroblast_Tmem119	Normal
Sptssb	5.80E-05	0.222392421	0.167	0	0.869547578	Fibroblast_Tmem119	Normal
Synpo2	5.80E-05	0.222392421	0.167	0	0.869547578	Fibroblast_Tmem119	Normal
Tbc1d9b	5.80E-05	0.222392421	0.167	0	0.869547578	Fibroblast_Tmem119	Normal
Tgfbr3	5.80E-05	0.222392421	0.167	0	0.869547578	Fibroblast_Tmem119	Normal
Thbs4	5.80E-05	0.222392421	0.167	0	0.869547578	Fibroblast_Tmem119	Normal
Thrsp	5.80E-05	0.222392421	0.167	0	0.869547578	Fibroblast_Tmem119	Normal
Tmbim1	5.80E-05	0.222392421	0.167	0	0.869547578	Fibroblast_Tmem119	Normal
Tmem29	5.80E-05	0.222392421	0.167	0	0.869547578	Fibroblast_Tmem119	Normal
Tmem47	5.80E-05	0.222392421	0.167	0	0.869547578	Fibroblast_Tmem119	Normal
Tpp2	5.80E-05	0.222392421	0.167	0	0.869547578	Fibroblast_Tmem119	Normal
Trmt1l	5.80E-05	0.222392421	0.167	0	0.869547578	Fibroblast_Tmem119	Normal
Ttc13	5.80E-05	0.222392421	0.167	0	0.869547578	Fibroblast_Tmem119	Normal
Txnl1	5.80E-05	0.222392421	0.167	0	0.869547578	Fibroblast_Tmem119	Normal
Ugt2b5	5.80E-05	0.222392421	0.167	0	0.869547578	Fibroblast_Tmem119	Normal
Unc13d	5.80E-05	0.222392421	0.167	0	0.869547578	Fibroblast_Tmem119	Normal
Unc93b1	5.80E-05	0.222392421	0.167	0	0.869547578	Fibroblast_Tmem119	Normal
Vamp1	5.80E-05	0.222392421	0.167	0	0.869547578	Fibroblast_Tmem119	Normal
Vps52	5.80E-05	0.222392421	0.167	0	0.869547578	Fibroblast_Tmem119	Normal
Wdr18	5.80E-05	0.222392421	0.167	0	0.869547578	Fibroblast_Tmem119	Normal
Zfp809	5.80E-05	0.222392421	0.167	0	0.869547578	Fibroblast_Tmem119	Normal
Zfp869	5.80E-05	0.222392421	0.167	0	0.869547578	Fibroblast_Tmem119	Normal
Zmym6	5.80E-05	0.222392421	0.167	0	0.869547578	Fibroblast_Tmem119	Normal

Manf	6.46E-05	0.513879403	0.5	0.051	0.968509926	Fibroblast_Tmem119	Normal
Malat1	0.000255164	0.486398666	0.5	0.061	1	Fibroblast_Tmem119	Normal
Nedd8	0.000927865	0.343954401	0.333	0.03	1	Fibroblast_Tmem119	Normal
Cyp3a11	0.00297668	0.357893592	0.333	0.04	1	Fibroblast_Tmem119	Normal
Rps25	0.003334474	0.343954401	0.333	0.04	1	Fibroblast_Tmem119	Normal
Rpl24	0.006101596	0.406964199	0.5	0.101	1	Fibroblast_Tmem119	Normal
Cldnd1	0.006705134	0.722466024	0.167	0.01	1	Fibroblast_Tmem119	Normal
Ddx1	0.006705134	0.40053793	0.167	0.01	1	Fibroblast_Tmem119	Normal
Kctd10	0.006705134	0.40053793	0.167	0.01	1	Fibroblast_Tmem119	Normal
Eef1a1	0.006937585	0.75161237	0.833	0.374	1	Fibroblast_Tmem119	Normal
Ahnak	0.007315794	0.207892852	0.167	0.01	1	Fibroblast_Tmem119	Normal
Amotl2	0.007315794	0.207892852	0.167	0.01	1	Fibroblast_Tmem119	Normal
Arf3	0.007315794	0.207892852	0.167	0.01	1	Fibroblast_Tmem119	Normal
Atp6v1g1	0.007315794	0.207892852	0.167	0.01	1	Fibroblast_Tmem119	Normal
Bub3	0.007315794	0.207892852	0.167	0.01	1	Fibroblast_Tmem119	Normal
Ccnd2	0.007315794	0.207892852	0.167	0.01	1	Fibroblast_Tmem119	Normal
Cd200	0.007315794	0.207892852	0.167	0.01	1	Fibroblast_Tmem119	Normal
Col5a3	0.007315794	0.207892852	0.167	0.01	1	Fibroblast_Tmem119	Normal
Cox6b1	0.007315794	0.207892852	0.167	0.01	1	Fibroblast_Tmem119	Normal
Crp	0.007315794	0.207892852	0.167	0.01	1	Fibroblast_Tmem119	Normal
Cyhr1	0.007315794	0.207892852	0.167	0.01	1	Fibroblast_Tmem119	Normal
Fam160b2	0.007315794	0.207892852	0.167	0.01	1	Fibroblast_Tmem119	Normal
Gpn1	0.007315794	0.207892852	0.167	0.01	1	Fibroblast_Tmem119	Normal
Gps1	0.007315794	0.207892852	0.167	0.01	1	Fibroblast_Tmem119	Normal
Hdac1	0.007315794	0.207892852	0.167	0.01	1	Fibroblast_Tmem119	Normal
Iqgap2	0.007315794	0.207892852	0.167	0.01	1	Fibroblast_Tmem119	Normal
Irf9	0.007315794	0.207892852	0.167	0.01	1	Fibroblast_Tmem119	Normal

Kansl2	0.0073157 94	0.207892852	0.167	0.01	1	Fibroblast_Tmem119	Normal
Mars	0.0073157 94	0.207892852	0.167	0.01	1	Fibroblast_Tmem119	Normal
Mrps16	0.0073157 94	0.207892852	0.167	0.01	1	Fibroblast_Tmem119	Normal
Mrps6	0.0073157 94	0.207892852	0.167	0.01	1	Fibroblast_Tmem119	Normal
Mtpap	0.0073157 94	0.207892852	0.167	0.01	1	Fibroblast_Tmem119	Normal
Ndufa2	0.0073157 94	0.207892852	0.167	0.01	1	Fibroblast_Tmem119	Normal
Ndufb2	0.0073157 94	0.207892852	0.167	0.01	1	Fibroblast_Tmem119	Normal
Net1	0.0073157 94	0.207892852	0.167	0.01	1	Fibroblast_Tmem119	Normal
Nfx1	0.0073157 94	0.207892852	0.167	0.01	1	Fibroblast_Tmem119	Normal
Nox4	0.0073157 94	0.207892852	0.167	0.01	1	Fibroblast_Tmem119	Normal
Npepps	0.0073157 94	0.207892852	0.167	0.01	1	Fibroblast_Tmem119	Normal
Npr2	0.0073157 94	0.207892852	0.167	0.01	1	Fibroblast_Tmem119	Normal
Prdm2	0.0073157 94	0.207892852	0.167	0.01	1	Fibroblast_Tmem119	Normal
Psmc3	0.0073157 94	0.207892852	0.167	0.01	1	Fibroblast_Tmem119	Normal
Ptch1	0.0073157 94	0.207892852	0.167	0.01	1	Fibroblast_Tmem119	Normal
Ptpn14	0.0073157 94	0.207892852	0.167	0.01	1	Fibroblast_Tmem119	Normal
Pum1	0.0073157 94	0.207892852	0.167	0.01	1	Fibroblast_Tmem119	Normal
Rad21	0.0073157 94	0.207892852	0.167	0.01	1	Fibroblast_Tmem119	Normal
Rgs19	0.0073157 94	0.207892852	0.167	0.01	1	Fibroblast_Tmem119	Normal
Rnf167	0.0073157 94	0.207892852	0.167	0.01	1	Fibroblast_Tmem119	Normal
Sec61b	0.0073157 94	0.207892852	0.167	0.01	1	Fibroblast_Tmem119	Normal
Serpina1e	0.0073157 94	0.207892852	0.167	0.01	1	Fibroblast_Tmem119	Normal
Slc16a2	0.0073157 94	0.207892852	0.167	0.01	1	Fibroblast_Tmem119	Normal
Slc24a3	0.0073157 94	0.207892852	0.167	0.01	1	Fibroblast_Tmem119	Normal
Slc25a36	0.0073157 94	0.207892852	0.167	0.01	1	Fibroblast_Tmem119	Normal
Socs7	0.0073157 94	0.207892852	0.167	0.01	1	Fibroblast_Tmem119	Normal
Ssb	0.0073157 94	0.207892852	0.167	0.01	1	Fibroblast_Tmem119	Normal

Szrd1	0.007315794	0.207892852	0.167	0.01	1	Fibroblast_Tmem119	Normal
Tank	0.007315794	0.207892852	0.167	0.01	1	Fibroblast_Tmem119	Normal
Timm8b	0.007315794	0.207892852	0.167	0.01	1	Fibroblast_Tmem119	Normal
Tm6sf2	0.007315794	0.207892852	0.167	0.01	1	Fibroblast_Tmem119	Normal
Tmem127	0.007315794	0.207892852	0.167	0.01	1	Fibroblast_Tmem119	Normal
Tpp1	0.007315794	0.207892852	0.167	0.01	1	Fibroblast_Tmem119	Normal
Tra2b	0.007315794	0.207892852	0.167	0.01	1	Fibroblast_Tmem119	Normal
Tram1	0.007315794	0.207892852	0.167	0.01	1	Fibroblast_Tmem119	Normal
Tyms	0.007315794	0.207892852	0.167	0.01	1	Fibroblast_Tmem119	Normal
Uba1	0.007315794	0.207892852	0.167	0.01	1	Fibroblast_Tmem119	Normal
Uqcrc2	0.007315794	0.207892852	0.167	0.01	1	Fibroblast_Tmem119	Normal
Vmn2r102	0.007315794	0.207892852	0.167	0.01	1	Fibroblast_Tmem119	Normal
Ybx3	0.007315794	0.207892852	0.167	0.01	1	Fibroblast_Tmem119	Normal
Hmgn1	0.007631537	0.343954401	0.333	0.051	1	Fibroblast_Tmem119	Normal
Muc2	0.007631537	0.343954401	0.333	0.051	1	Fibroblast_Tmem119	Normal
Cbx7	0.007981756	0.193537559	0.167	0.01	1	Fibroblast_Tmem119	Normal
Cul1	0.007981756	0.193537559	0.167	0.01	1	Fibroblast_Tmem119	Normal
Dnpep	0.007981756	0.193537559	0.167	0.01	1	Fibroblast_Tmem119	Normal
Rab5b	0.007981756	0.193537559	0.167	0.01	1	Fibroblast_Tmem119	Normal
Tex264	0.007981756	0.193537559	0.167	0.01	1	Fibroblast_Tmem119	Normal
Tmem132b	0.007981756	0.193537559	0.167	0.01	1	Fibroblast_Tmem119	Normal
Pdia3	0.007981756	0.179323699	0.167	0.01	1	Fibroblast_Tmem119	Normal
Myl6	0.008007099	0.663572335	0.667	0.202	1	Fibroblast_Tmem119	Normal

Naa10	5.94E-17	0.222392421	0.167	0	8.92E-13	Macrophage_Gpnmb	Normal
Fnta	1.57E-11	0.218961516	0.167	0.002	2.36E-07	Macrophage_Gpnmb	Normal
Gramd1a	1.57E-11	0.218961516	0.167	0.002	2.36E-07	Macrophage_Gpnmb	Normal
Ugt2b35	8.41E-09	0.21553875	0.167	0.005	0.000126139	Macrophage_Gpnmb	Normal
Ergic1	1.03E-07	0.391190757	0.25	0.017	0.001550409	Macrophage_Gpnmb	Normal

Gja1	1.23E-07	0.298081353	0.25	0.017	0.001841437	Macrophage_Gpnmb	Normal
Pcna	6.21E-07	0.291339775	0.25	0.019	0.009315055	Macrophage_Gpnmb	Normal
Bad	2.96E-05	0.205318908	0.167	0.012	0.444070433	Macrophage_Gpnmb	Normal
Eif3i	2.96E-05	0.205318908	0.167	0.012	0.444070433	Macrophage_Gpnmb	Normal
Aoc3	0.000117563	0.201928319	0.167	0.014	1	Macrophage_Gpnmb	Normal
Emp3	0.000117563	0.201928319	0.167	0.014	1	Macrophage_Gpnmb	Normal
Hmgcs2	0.000345813	0.198545679	0.167	0.017	1	Macrophage_Gpnmb	Normal
Flna	0.000348776	0.368122918	0.417	0.095	1	Macrophage_Gpnmb	Normal
Ddb1	0.000823744	0.195170953	0.167	0.019	1	Macrophage_Gpnmb	Normal
F2	0.000823744	0.195170953	0.167	0.019	1	Macrophage_Gpnmb	Normal
Arcn1	0.001682248	0.191804102	0.167	0.021	1	Macrophage_Gpnmb	Normal
Sri	0.001732798	0.188445089	0.167	0.021	1	Macrophage_Gpnmb	Normal
Cyp3a11	0.003059733	0.188445089	0.167	0.024	1	Macrophage_Gpnmb	Normal
Aldh1a1	0.003143511	0.18509388	0.167	0.024	1	Macrophage_Gpnmb	Normal
Map1lc3a	0.005089263	0.18509388	0.167	0.026	1	Macrophage_Gpnmb	Normal
Psmb4	0.005089263	0.18509388	0.167	0.026	1	Macrophage_Gpnmb	Normal
Rps27	0.005089263	0.18509388	0.167	0.026	1	Macrophage_Gpnmb	Normal
Ugt2b34	0.005089263	0.18509388	0.167	0.026	1	Macrophage_Gpnmb	Normal
Rpl36	0.007889031	0.181750437	0.167	0.029	1	Macrophage_Gpnmb	Normal
Alb	0.008107096	0.410464769	0.833	0.526	1	Macrophage_Gpnmb	Normal

Mt1	0.000410161	0.127195684	0.114	0.02	1	Macrophage_Ctsb	Normal
Ctsb	0.000804931	0.266466376	0.929	0.795	1	Macrophage_Ctsb	AAA

Hadh	1.49E-08	0.138871656	0.125	0.027	0.000224079	Adipose	Normal
Ndufb8	3.57E-08	0.145569638	0.14	0.034	0.000535462	Adipose	Normal
Aco2	1.35E-07	0.227484811	0.199	0.068	0.002030944	Adipose	Normal
Acadl	5.84E-06	0.16658805	0.118	0.034	0.087654108	Adipose	Normal

Ech1	8.80E-06	0.12748823	0.162	0.058	0.131981996	Adipose	Normal
Pnpla2	1.39E-05	0.219437868	0.191	0.079	0.209255841	Adipose	Normal
Atp5b	5.64E-05	0.163462826	0.162	0.065	0.845597294	Adipose	Normal
Ifi27	9.73E-05	0.121858193	0.118	0.041	1	Adipose	Normal
Pck1	0.00012401	0.140404118	0.132	0.05	1	Adipose	Normal
Cox6a1	0.000322542	0.132534451	0.235	0.121	1	Adipose	Normal
Glul	0.001496448	0.169084893	0.25	0.148	1	Adipose	Normal
Cs	0.001802519	0.082407462	0.103	0.042	1	Adipose	Normal
Cyb5a	0.003501812	0.096937513	0.118	0.054	1	Adipose	Normal
Cox4i1	0.004820212	0.125764374	0.176	0.099	1	Adipose	Normal
Car3	0.007353408	0.172795159	0.265	0.176	1	Adipose	Normal
Hp	8.61E-08	0.396772871	0.432	0.199	0.001291491	Adipose	AAA
Cfd	7.62E-05	0.377741765	0.901	0.868	1	Adipose	AAA
Col3a1	0.001162423	0.186031836	0.291	0.162	1	Adipose	AAA
Rpl13a	0.002898113	0.14459972	0.212	0.103	1	Adipose	AAA
C4b	0.003496156	0.137305132	0.119	0.037	1	Adipose	AAA

Supplemental Table 4. Sequences of the primers used for real-time PCR

	Primer Sequence	
mouse *Gpnmb*	Forward	ACGGCAGGTGGAAGGACT
	Reverse	CGGTGAGTCACTGGTCAGG
mouse *Actb*	Forward	GTGACGTTGACATCCGTAAAGA
	Reverse	GCCGGACTCATCGTACTCC
human *GPNMB*	Forward	TAAACCTTGAGTGCCTGCGTC
	Reverse	GTCTTTCATTGCCCAGCACA
human *CD44*	Forward	TCTACCCCAGCAACCCTACT
	Reverse	TTGGTTGCTGTCTCAGTTGCT
human *EGFR*	Forward	TCCAGTATTGATCGGGAGAGCC
	Reverse	TGAGCTTGTTACTCGTGCCTT
human *KDR*	Forward	CAAGTGGCTAAGGGCATGGA
	Reverse	ATTTCAAAGGGAGGCGAGCA
human *MYH11*	Forward	CATCTACTCGGAGAAGATCGTCG
	Reverse	CGCCTGTGCATAGAATGGACT
human *ACTA2*	Forward	CTTGAGAAGAGTTACGAGTTG
	Reverse	GATGCTGTTGTAGGTGGTT
human *TAGLN*	Forward	AGTGCAGTCCAAAATCGAGAAG
	Reverse	CTTGCTCAGAATCACGCCAT
human *CCL2*	Forward	CAGCCAGATGCAATCAATGCC
	Reverse	TGGAATCCTGAACCCACTTCT
human *IL6*	Forward	ACTCACCTCTTCAGAACGAATTG
	Reverse	CCATCTTTGGAAGGTTCAGGTTG
human *CXCL3*	Forward	CGCCCAAACCGAAGTCATAG
	Reverse	GCTCCCCTTGTTCAGTATCTTTT
human *CXCL5*	Forward	AGCTGCGTTGCGTTTGTTTAC
	Reverse	TGGCGAACACTTGCAGATTAC
human *MMP2*	Forward	TACAGGATCATTGGCTACACACC
	Reverse	GGTCACATCGCTCCAGACT
human *MMP9*	Forward	AGACCTGGGCAGATTCCAAAC
	Reverse	CGGCAAGTCTTCCGAGTAGT
human *ADAM10*	Forward	AGCAACATCTGGGGACAAAC
	Reverse	CCCAGGTTTCAGTTTGCATT
human *ACTB*	Forward	CATGTACGTTGCTATCCAGGC
	Reverse	CTCCTTAATGTCACGCACGAT

Major Resource Table

Animals

Species (Mouse)	Source	Stock No.	Sex
$Apoe^{-/-}$	The Jackson Lab	002052	male
C57BL/6J	The Jackson Lab	000664	male

Antibodies

**Target antigen**	**Source**	**Catalog No.**	**Applications**	**Working concentration**
GPNMB	Fisher Scientific	AF2330	IF	2μg/ml
Mac2	Thermo Fisher Scientific	14-5301-85	IF	5μg/ml
α-actin	Abcam	Ab5694	IF	1μg/ml
CD44	Fisher Scientific	5012580	IF	5μg/ml
SM22α	Abcam	ab10135	IF&WB	IF, 2.5μg/ml; WB, 0.5μg/ml
α-actin	Abcam	ab119952	WB	0.5μg/ml

IF, immunofluorescence staining; WB, western blot.
