# Supplementary material for: Single-cell spatial transcriptomics unravels the cellular landscape of abdominal aortic aneurysm
Source: JCI Insight. 2025 Aug 22;10(16):e190534. doi: 10.1172/jci.insight.190534 (PMC12406718; doi:10.1172/jci.insight.190534)
Supplement: Unedited blot and gel images [file jciinsight-10-190534-s267.pdf]

Uncropped western blots

Single-Cell Spatial Transcriptomics Unravels the Cellular Landscape of Abdominal Aortic Aneurysm

The red boxes label the representative blots in the figures

Figure 4C

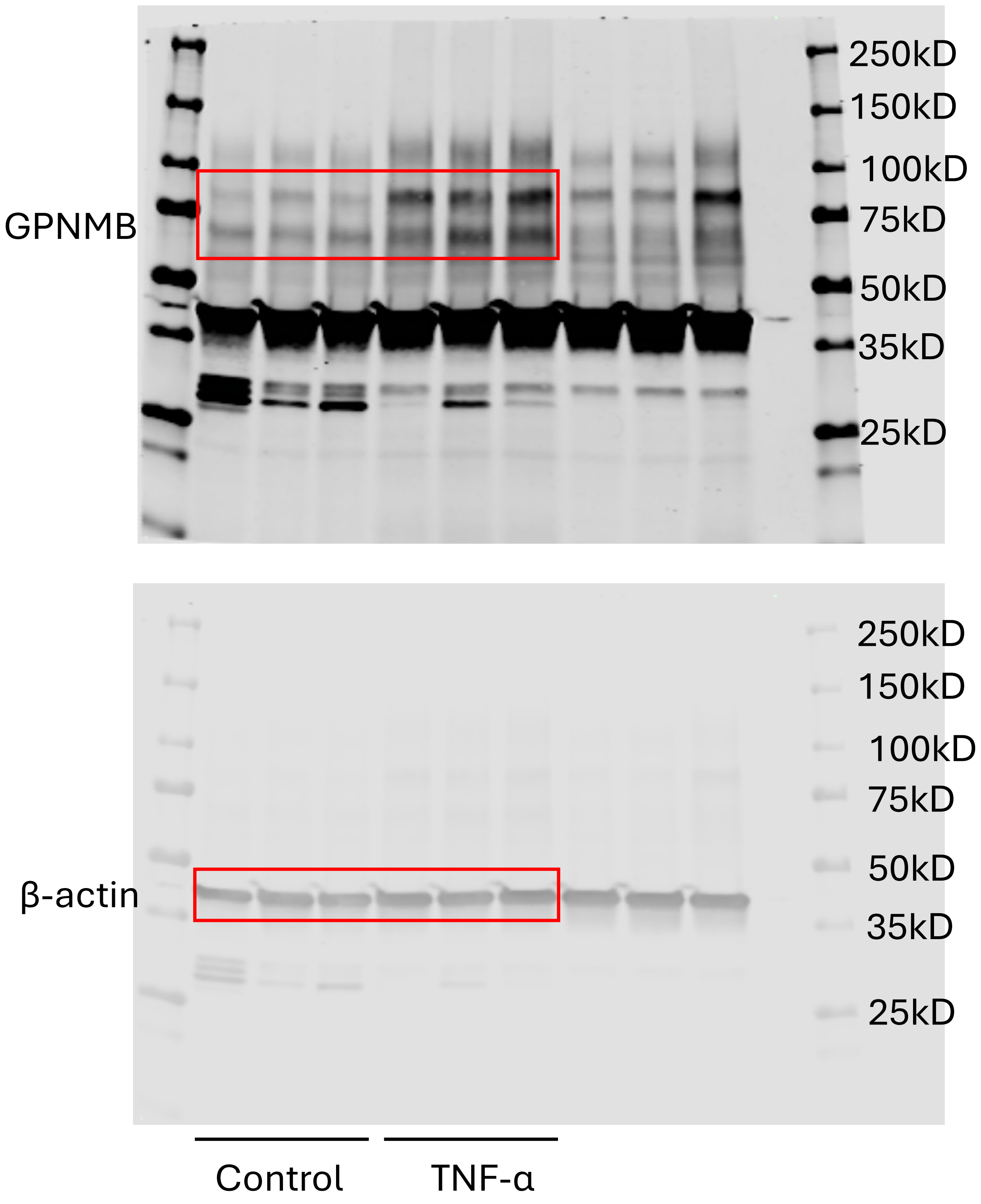
GPNMB
250kD
150kD
100kD
75kD
50kD
35kD
25kD
β-actin
250kD
150kD
100kD
75kD
50kD
35kD
25kD
Control
TNF-α

Figure 4G

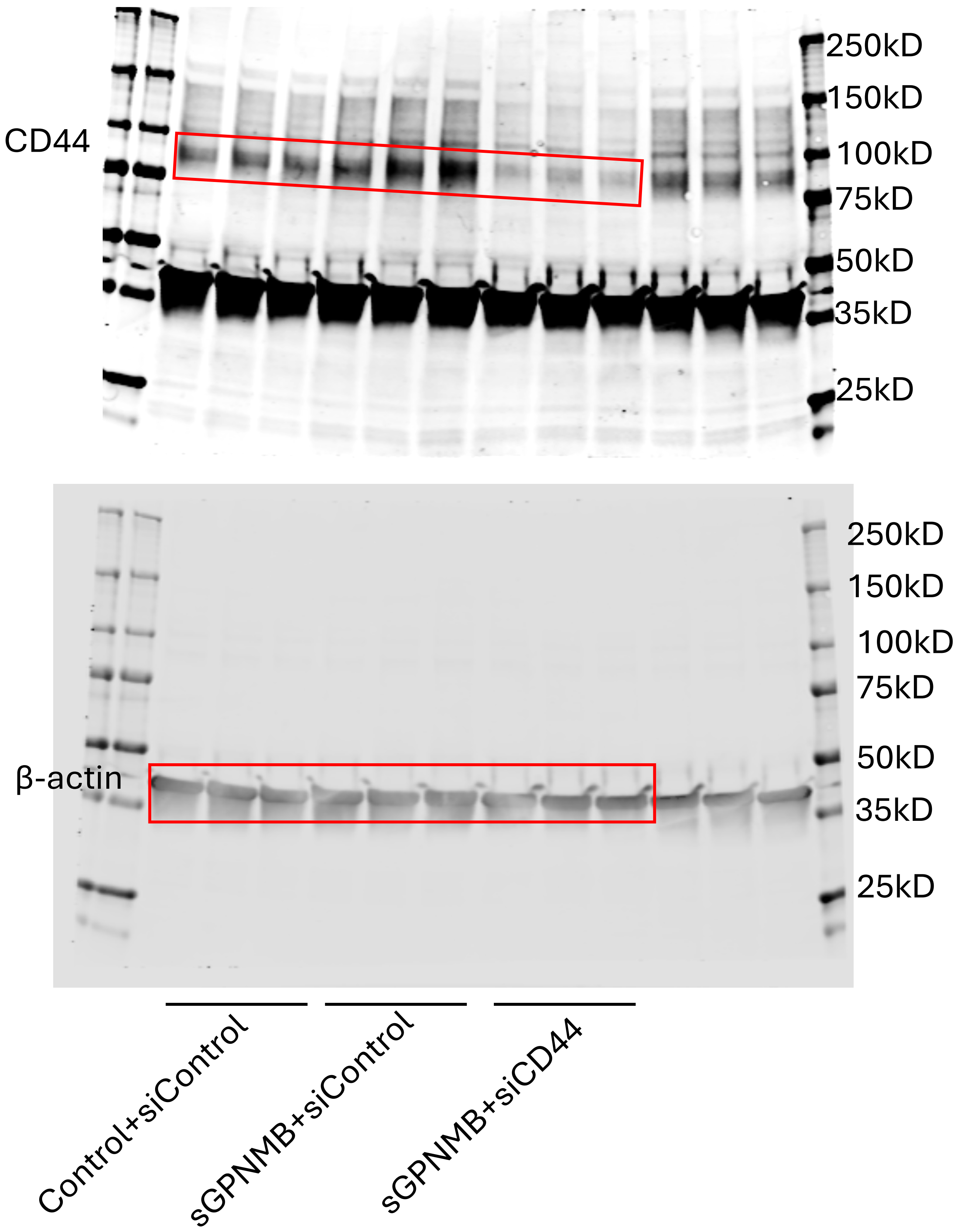

CD44
250kD
150kD
100kD
75kD
50kD
35kD
25kD
β-actin
250kD
150kD
100kD
75kD
50kD
35kD
25kD
Control+siControl
sGPNMB+siControl
sGPNMB+siCD44

Figure 4I

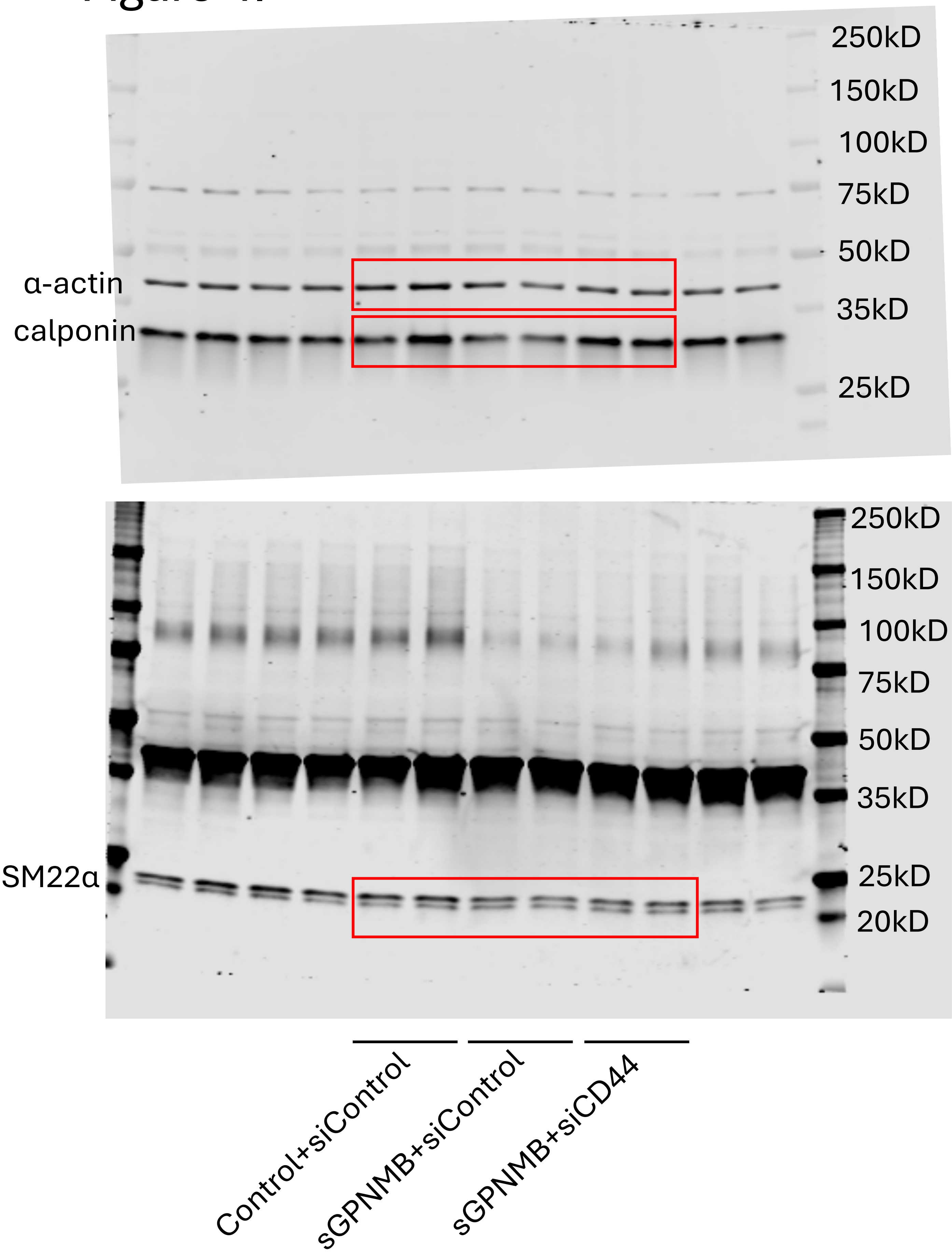

250kD
150kD
100kD
75kD
50kD
α-actin
calponin
35kD
25kD
250kD
150kD
100kD
75kD
50kD
35kD
SM22α
25kD
20kD
Control+siControl
sGPNMB+siControl
sGPNMB+siCD44

Figure 4I

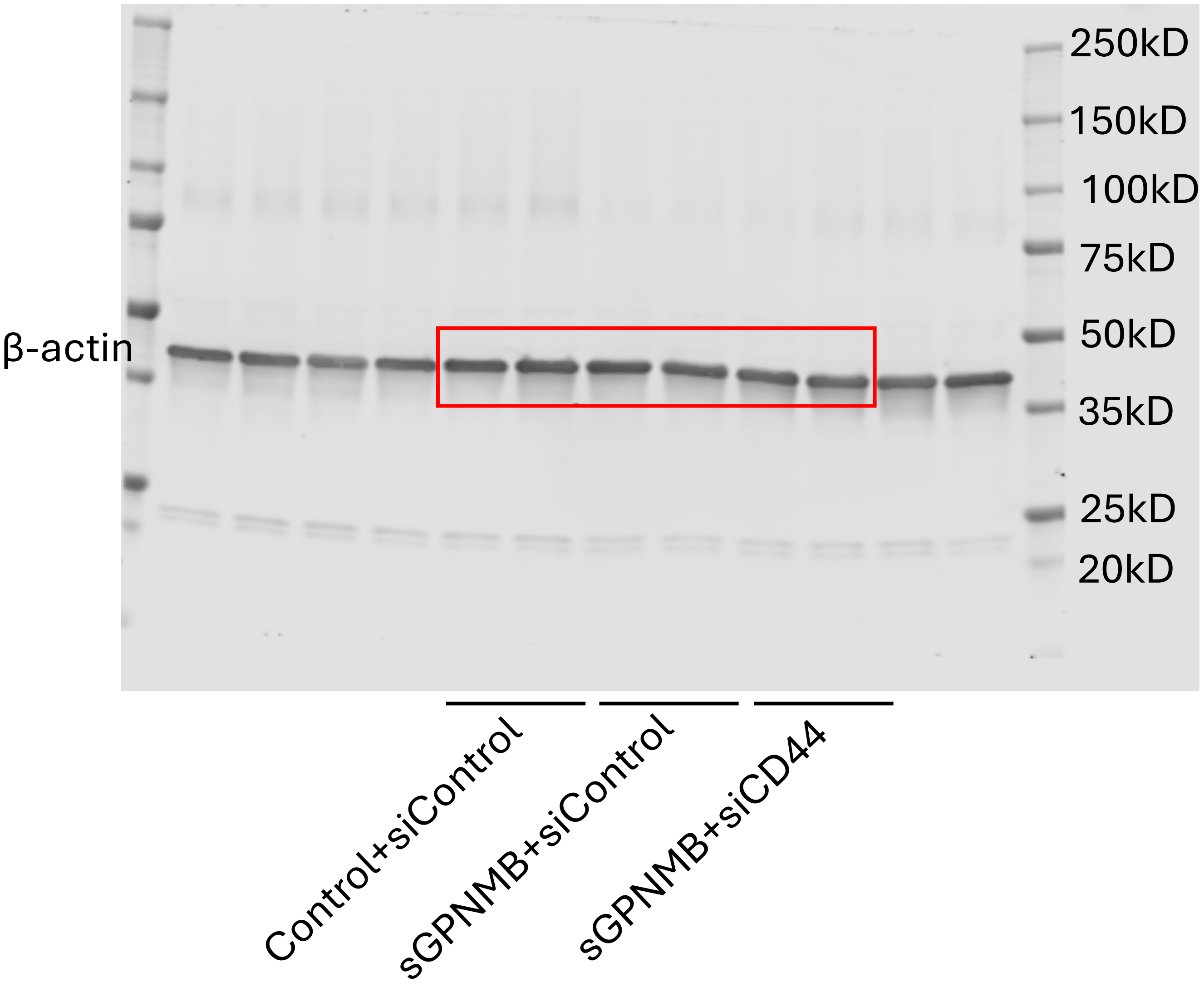

β-actin
250kD
150kD
100kD
75kD
50kD
35kD
25kD
20kD
Control+siControl
sGPNMB+siControl
sGPNMB+siCD44

Figure S8

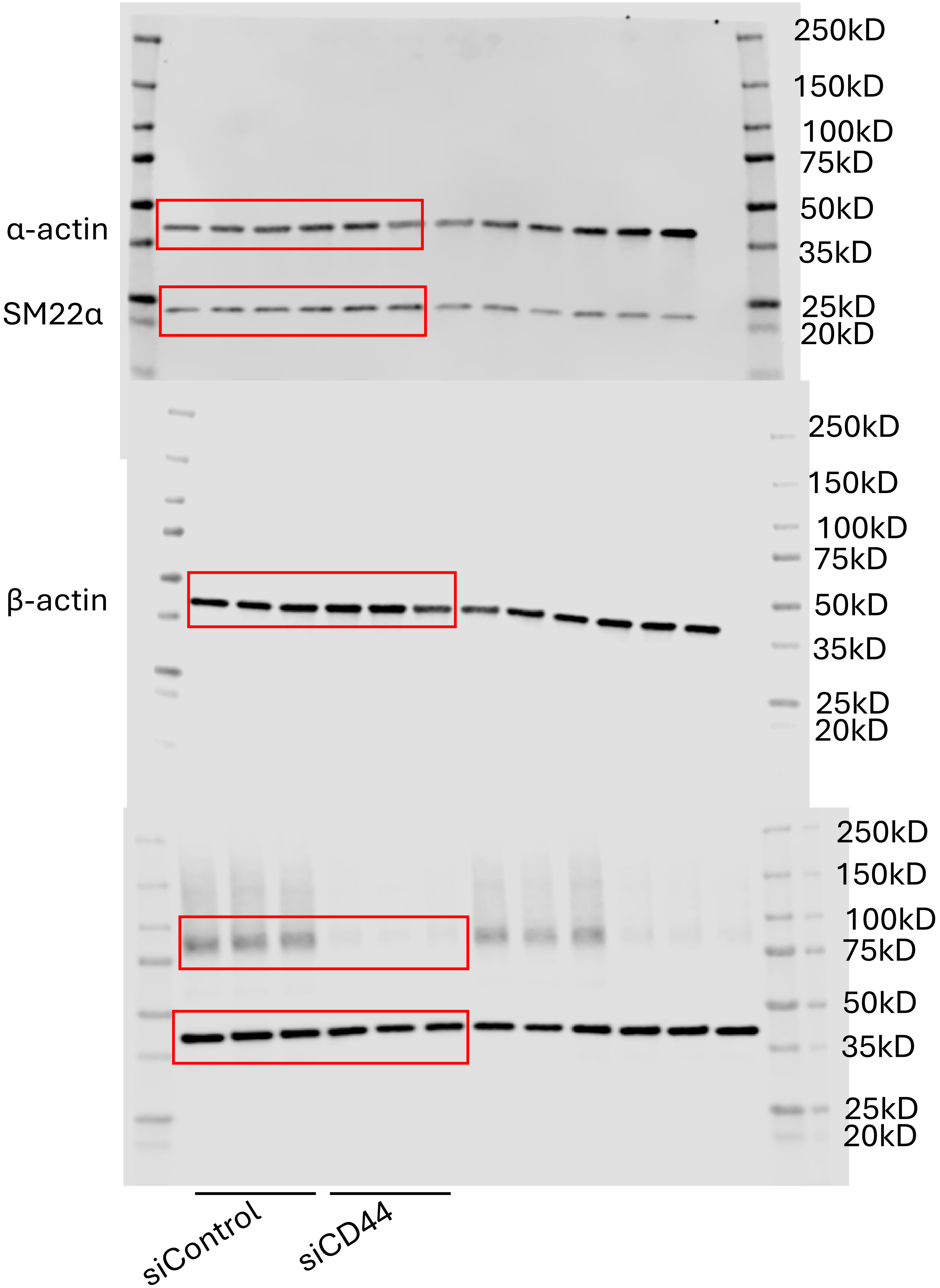

α-actin
SM22α
β-actin
250kD
150kD
100kD
75kD
50kD
35kD
25kD
20kD
siControl
siCD44
